# COVID-19 in pregnant women: a systematic review and meta-analysis on the risk and prevalence of pregnancy loss

**DOI:** 10.1093/humupd/dmad030

**Published:** 2023-11-28

**Authors:** Janneke A C van Baar, Elena B Kostova, John Allotey, Shakila Thangaratinam, Javier R Zamora, Mercedes Bonet, Caron Rahn Kim, Lynne M Mofenson, Heinke Kunst, Asma Khalil, Elisabeth van Leeuwen, Julia Keijzer, Marije Strikwerda, Bethany Clark, Maxime Verschuuren, Arri Coomarasamy, Mariëtte Goddijn, Madelon van Wely, PregCOV-19 Living Systematic Review Consortium, Elena Stallings, Magnus Yap, Shaunak Chatterjee, Tania Kew, Luke Debenham, Anna Clavé Llavall, Anushka Dixit, Dengyi Zhou, Rishab Balaji, Xiu Qiu, Mingyang Yuan, Dyuti Coomar, Siang Ing Lee, Vanessa Brizuela, Nathalie Jeanne Nicole Broutet, Edna Kara, Caron Rahn Kim, Anna Thorson, Olufemi Taiwo Oladapo

**Affiliations:** Department of Obstetrics and Gynaecology, Center for Reproductive Medicine, Amsterdam UMC, Amsterdam, The Netherlands; Department of Obstetrics and Gynaecology, Center for Reproductive Medicine, Amsterdam UMC, Amsterdam, The Netherlands; Cochrane Gynaecology and Fertility Satellite, Amsterdam, The Netherlands; Amsterdam Reproduction and Development Research Institute, Amsterdam, The Netherlands; Birmingham Women’s and Children’s NHS Foundation Trust, Birmingham, UK; NIHR Biomedical Research Center, University Hospitals Birmingham, Birmingham, UK; Birmingham Women’s and Children’s NHS Foundation Trust, Birmingham, UK; NIHR Biomedical Research Center, University Hospitals Birmingham, Birmingham, UK; WHO Collaborating Centre for Global Women’s Health, Institute of Metabolism and Systems Research, University of Birmingham, Birmingham, UK; WHO Collaborating Centre for Global Women’s Health, Institute of Metabolism and Systems Research, University of Birmingham, Birmingham, UK; Clinical Biostatistics Unit, Hospital Universitario Ramón y Cajal (IRYCIS), Madrid, Spain; CIBER Epidemiology and Public Health (CIBERESP), Madrid, Spain; UNDP/UNFPA/UNICEF/WHO/World Bank Special Programme of Research, Development and Research Training in Human Reproduction (HRP), Department of Sexual and Reproductive Health and Research, World Health Organization, Geneva, Switzerland; UNDP/UNFPA/UNICEF/WHO/World Bank Special Programme of Research, Development and Research Training in Human Reproduction (HRP), Department of Sexual and Reproductive Health and Research, World Health Organization, Geneva, Switzerland; Elizabeth Glaser Pediatric AIDS Foundation, Washington, DC, USA; Blizard Institute, Barts and The London School of Medicine and Dentistry, Queen Mary University of London, London, UK; Barts Health NHS Trust, London, UK; St George’s University London, London, UK; Amsterdam Reproduction and Development Research Institute, Amsterdam, The Netherlands; Women and Childrens Hospital, Amsterdam UMC, Amsterdam, The Netherlands; Department of Obstetrics and Gynaecology, Center for Reproductive Medicine, Amsterdam UMC, Amsterdam, The Netherlands; Department Vrouw & Baby, Utrecht UMC, location University of Utrecht, Utrecht, The Netherlands; Department Vrouw & Baby, Utrecht UMC, location University of Utrecht, Utrecht, The Netherlands; Department of Obstetrics and Gynaecology, Center for Reproductive Medicine, Amsterdam UMC, Amsterdam, The Netherlands; Birmingham Women’s and Children’s NHS Foundation Trust, Birmingham, UK; NIHR Biomedical Research Center, University Hospitals Birmingham, Birmingham, UK; Tommy's Centre for Miscarriage Research, Birmingham, UK; Department of Obstetrics and Gynaecology, Center for Reproductive Medicine, Amsterdam UMC, Amsterdam, The Netherlands; Amsterdam Reproduction and Development Research Institute, Amsterdam, The Netherlands; Department of Obstetrics and Gynaecology, Center for Reproductive Medicine, Amsterdam UMC, Amsterdam, The Netherlands; Cochrane Gynaecology and Fertility Satellite, Amsterdam, The Netherlands; Amsterdam Reproduction and Development Research Institute, Amsterdam, The Netherlands

**Keywords:** SARS-CoV-2, severe acute respiratory syndrome coronavirus 2, COVID-19, coronavirus disease 2019, miscarriage, (early) pregnancy loss, ectopic pregnancy, abortion, spontaneous abortion, termination of pregnancy

## Abstract

**BACKGROUND:**

Pregnant women infected with severe acute respiratory syndrome coronavirus 2 (SARS-CoV-2) are more likely to experience preterm birth and their neonates are more likely to be stillborn or admitted to a neonatal unit. The World Health Organization declared in May 2023 an end to the coronavirus disease 2019 (COVID-19) pandemic as a global health emergency. However, pregnant women are still becoming infected with SARS-CoV-2 and there is limited information available regarding the effect of SARS-CoV-2 infection in early pregnancy on pregnancy outcomes.

**OBJECTIVE AND RATIONALE:**

We conducted this systematic review to determine the prevalence of early pregnancy loss in women with SARS-Cov-2 infection and compare the risk to pregnant women without SARS-CoV-2 infection.

**SEARCH METHODS:**

Our systematic review is based on a prospectively registered protocol. The search of PregCov19 consortium was supplemented with an extra electronic search specifically on pregnancy loss in pregnant women infected with SARS-CoV-2 up to 10 March 2023 in PubMed, Google Scholar, and LitCovid. We included retrospective and prospective studies of pregnant women with SARS-CoV-2 infection, provided that they contained information on pregnancy losses in the first and/or second trimester. Primary outcome was miscarriage defined as a pregnancy loss before 20 weeks of gestation, however, studies that reported loss up to 22 or 24 weeks were also included. Additionally, we report on studies that defined the pregnancy loss to occur at the first and/or second trimester of pregnancy without specifying gestational age, and for second trimester miscarriage only when the study presented stillbirths and/or foetal losses separately from miscarriages. Data were stratified into first and second trimester. Secondary outcomes were ectopic pregnancy (any extra-uterine pregnancy), and termination of pregnancy. At least three researchers independently extracted the data and assessed study quality. We calculated odds ratios (OR) and risk differences (RDs) with corresponding 95% CI and pooled the data using random effects meta-analysis. To estimate risk prevalence, we performed meta-analysis on proportions. Heterogeneity was assessed by *I*^2^.

**OUTCOMES:**

We included 120 studies comprising a total of 168 444 pregnant women with SARS-CoV-2 infection; of which 18 233 women were in their first or second trimester of pregnancy. Evidence level was considered to be of low to moderate certainty, mostly owing to selection bias. We did not find evidence of an association between SARS-CoV-2 infection and miscarriage (OR 1.10, 95% CI 0.81–1.48; *I*^2^ = 0.0%; RD 0.0012, 95% CI −0.0103 to 0.0127; *I*^2^ = 0%; 9 studies, 4439 women). Miscarriage occurred in 9.9% (95% CI 6.2–14.0%; *I*^2^ = 68%; 46 studies, 1797 women) of the women with SARS CoV-2 infection in their first trimester and in 1.2% (95% CI 0.3–2.4%; *I*^2^ = 34%; 33 studies; 3159 women) in the second trimester. The proportion of ectopic pregnancies in women with SARS-CoV-2 infection was 1.4% (95% CI 0.02–4.2%; *I*^2^ = 66%; 14 studies, 950 women). Termination of pregnancy occurred in 0.6% of the women (95% CI 0.01–1.6%; *I*^2^ = 79%; 39 studies; 1166 women).

**WIDER IMPLICATIONS:**

Our study found no indication that SARS-CoV-2 infection in the first or second trimester increases the risk of miscarriages. To provide better risk estimates, well-designed studies are needed that include pregnant women with and without SARS-CoV-2 infection at conception and early pregnancy and consider the association of clinical manifestation and severity of SARS-CoV-2 infection with pregnancy loss, as well as potential confounding factors such as previous pregnancy loss. For clinical practice, pregnant women should still be advised to take precautions to avoid risk of SARS-CoV-2 exposure and receive SARS-CoV-2 vaccination.

## Graphical abstract

**Figure:**
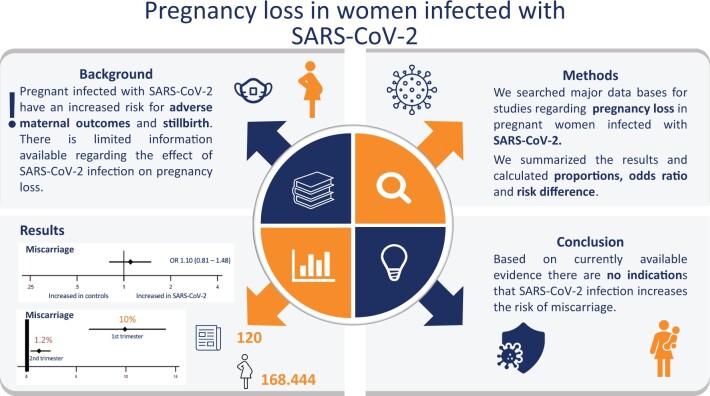
**An analysis of studies on the prevalence of early pregnancy loss in women infected with SARS-CoV-2 compared to pregnant women without SARS-CoV-2 infection showed no indications that SARS-CoV-2 infection increases risk of miscarriage.** SARS-CoV-2, severe acute respiratory syndrome coronavirus 2; OR, odds ratio.

## Introduction

Pregnant women infected with severe acute respiratory syndrome coronavirus 2 (SARS-CoV-2) have been shown to be at increased risk for severe coronavirus disease 2019 (COVID-19), with higher rates of pneumonia and respiratory failure, compared to non-pregnant women with SARS-CoV-2 infection. In addition, SARS-CoV-2 infected pregnant women also have an increased risk for adverse pregnancy outcomes compared to pregnant women without SARS-CoV-2 infection (Allotey *et al*., 2020; Ahmad *et al*., 2022). However, most data on SARS-CoV-2 infection in pregnancy stems from surveillance or research cohorts primarily including pregnant women infected with SARS-CoV-2 late in pregnancy; there is only limited information available regarding the effect of SARS-CoV-2 infection in early pregnancy and its relation to pregnancy outcome.

The PregCOV19 consortium is an international team of experts that aim to undertake living systematic reviews involving pregnant and postnatal women at risk, suspected, and diagnosed to have SARS-CoV-2 infection, and synthesize the relevant evidence on prevalence, risk factors, mother-to-child transmission, diagnosis, and treatment of the disease. The consortium began a living systematic review and meta-analysis in April 2020 to determine the clinical manifestations of SARS-CoV-2 infection in pregnant women, identify risk factors for complications, and quantify maternal and perinatal outcomes. The review found that pregnant women with SARS-CoV-2 infection are at an increased risk to deliver a stillborn child and to deliver preterm than pregnant women without SARS-CoV-2 infection (Allotey *et al*., 2020). Furthermore, venous thromboembolism and disseminated intravascular coagulation (DIC) have also been noted more frequently in pregnant women with SARS-CoV-2 infection than those without infection (Al-Samkari *et al*., 2020; Cruz Melguizo *et al*., 2021). DIC is a pathological disruption of the process of haemostasis and is a leading cause for maternal mortality, often secondary to underlying maternal/foetal complications, such as placental abruption, amniotic fluid embolism or HELLP (Haemolysis, Elevated Liver enzymes and Low Platelets) syndrome (Erez *et al*., 2022). Placental abnormalities related to maternal and foetal malperfusion, villous fibrin deposits, foetal vasculopathy, as well as inflammatory alterations have been described with SARS-CoV-2 infection in third trimester of pregnancy, which in some studies has been associated with increased risk of stillbirth (Joshi *et al*., 2022; Schwartz *et al*., 2022). The presence of such placental abnormalities associated with SARS-CoV-2 infection in early pregnancy might also result in higher miscarriage rates (Pabinger *et al*., 2001; Collins *et al*., 2022). Few studies have evaluated the question of whether SARS-CoV-2 infection in pregnant women during the first or second trimester of pregnancy might lead to pregnancy loss.

Viral infections during pregnancy have been linked with adverse pregnancy outcomes and birth defects (Racicot and Mor, 2017). Particular viruses can infect several cellular components of the placenta, while other viruses can directly infect the foetus at specific times during gestation. This increase in adverse maternal/foetal outcomes can especially be seen during pandemics such as influenza, Ebola, and Lassa fever (Silasi *et al*., 2015). Moreover, a recent study found that influenza during pregnancy is associated with pregnancy loss at >13 weeks of gestation and decreased infant birthweight, and that the risk of influenza is highest in the first trimester of pregnancy (Dawood *et al*., 2021). Since influenza and SARS-CoV-2 share immunopathological similarities, SARS-CoV-2 infection in early pregnancy might increase the risk of pregnancy loss as well (Khorramdelazad *et al*., 2021).

Early pregnancy loss includes miscarriages and ectopic pregnancies (EPs). A miscarriage is generally defined as the spontaneous loss of pregnancy before 20–22 weeks of gestation, though in some countries the definition includes pregnancy loss up to 24 weeks (Prager *et al*., 2023). Globally, an estimated 23 million miscarriages occur every year, affecting 1 in 10 women in their lifetime (Lancet, 2021). Most miscarriages (80%) occur before 12 weeks of gestation, while late miscarriages (usually between 12 and 20–22 weeks of gestation) are less frequent, with an estimated rate of 1–2% of all pregnancies (Practice Committee of the American Society for Reproductive Medicine, 2012; Dugas and Slane, 2021). EP occurs when the embryo implants outside the uterus, usually in one of the fallopian tubes, and occurs in an estimated 2% of pregnancies. SARS-CoV-2 infection should not affect development of EP, but some studies have described an increased rate of ruptured EP during the COVID-19 pandemic, possibly linked to delayed access to medical care during the pandemic (Casadio *et al*., 2020; Barg *et al*., 2021).

Even though the World Health Organization (WHO) has declared an end to the COVID-19 pandemic as a global health emergency, people are still becoming infected with SARS-CoV-2. This systematic review specifically aims to study the prevalence of pregnancy loss in pregnant women with confirmed SARS-CoV-2 infection and whether this differs compared to pregnant women without SARS-CoV-2 infection. We hypothesize that SARS-CoV-2 infection in pregnant women increases the chance of a first or second trimester miscarriage.

## Methods

Our systematic review is based on a prospectively registered protocol (PROSPERO CRD42020178076; registered 22 April 2020). For this project, a short separate protocol was developed (https://osf.io/e8dhr/).

### Search strategy

We used the PregCOV19 search, as described previously (Yap *et al*., 2020). Subsequently, an extra electronic literature search was conducted (in duplicate by J.v.B., J.K., M.S., M.V., B.C., E.K., and/or M.v.W.) specifically addressing pregnancy loss in pregnant women with COVID-19 up to 10 March 2023 in the following medical databases: PubMed, Google Scholar, and LitCovid (Supplementary Table S1). Finally, the reference lists of relevant studies were examined to identify additional studies.

### Study selection

We screened the queried articles on title and abstract for eligibility. All studies of pregnant women with confirmed or suspected SARS-CoV-2 infection were included, provided that they contained information on pregnancy loss (miscarriage and/or EP) or on termination of pregnancy (TOP). The cases were defined as pregnant women with SARS-CoV-2 infection who had a pregnancy loss preferably before 20 weeks of gestation, however, studies that reported loss up to 22 or 24 weeks were also included. Moreover, we included studies that defined pregnancy loss to occur at the first and/or second trimester of pregnancy without specifying gestational age. The control group was defined as pregnant and postpartum women without SARS-CoV-2 infection. Study groups used cohorts for sequential publications over time. To prevent multiple inclusion of the same data we selected the latest or largest study and excluded the overlapping studies. Studies were also excluded if they were non-peer reviewed papers, review articles, guidelines, and opinion pieces. When an overlap in data was expected, the study with most complete data was included.

Women were defined as having confirmed SARS-CoV-2 infection if they had confirmation through reverse transcription PCT (RT-PCR). Suspected SARS-CoV-2 infection was defined as women with a diagnosis based solely on clinical, serological, and radiological findings. We excluded studies when pregnancy loss data and/or having had SARS-CoV-2 infection was based on self-reporting.

### Data extraction and study quality assessment

A structured data-extraction form was used, and data were extracted by multiple reviewers (J.v.B., J.K., M.S., M.V., B.C., E.K., M.v.W.). The data-extraction sheet of the main search from the PregCov group located in Birmingham was shared (J.Z. and S.T.), and all data extracted were cross-checked. We went back to the original studies to recheck the data (by J.v.B., E.K., M.v.W.) in case of discrepancies or missing data. The following study design characteristics were extracted: the first author’s name, setting, year of publication, country of origin, and study design. The documented patient’s characteristics were total number of patients included, number of patients included with confirmed SARS-CoV-2 infection, age of patients in years, BMI in kg/m^2^, and other information about the patient spectrum including demographic characteristics, type of pregnancy loss, severity of COVID-19-related disease, previous miscarriages, smoking, and the week and/or trimester of gestation the pregnancy loss/termination occurred. The extracted data are also part of an open database (https://cgf.cochrane.org/news/covid-19-coronavirus-disease-fertility-and-pregnancy).

Methodological quality of included comparative cohort studies was assessed using the Newcastle-Ottawa Scale (Wells *et al*., 2000) for selection, comparability, and outcome ascertainment bias. As described previously (Allotey *et al*., 2020; Yap *et al*., 2020), studies achieving four stars for selection, two for comparability, and three for ascertainment of the outcome were considered to have a low risk of bias. Studies achieving two or three stars for selection, one for comparability, and two for outcome ascertainment were considered to have moderate risk of bias, and any study achieving one star for selection or outcome ascertainment, or zero for any of the three domains, was regarded as having a high risk of bias. The quality of prevalence studies was assessed using the validated tool by Hoy *et al*. (2012). The following domains were considered on risk of bias for external validity: population, sampling frame, selection, and non-response bias. The following domains were assessed on risk of bias for internal validity: data collection, case definition, reliability and validity, and mode of data collection, adequate follow up and appropriate numerator and denominator. The critical appraisal of included studies was carried out by three reviewers (J.v.B., E.K., M.v.W.).

GRADE was used to determine the certainty of the evidence; because all studies were observational the certainty of the evidence was initially set at ‘low’, with the possibility to be down or upgraded.

We excluded studies from the meta-analyses that reported on 10 or less cases, and those with 100% miscarriage in the first or second trimester in view of the selection bias. We excluded studies from the comparative meta-analysis when numerator and/or denominators were unclear and when SARS-CoV-2 infection was based on self-reporting.

### Outcomes and definitions

Primary outcome was miscarriage ≤20 weeks of gestation, however, studies that reported pregnancy loss up to 22 or 24 weeks were also included. Additionally, we included studies that defined the pregnancy loss to occur at the first and/or second trimester of pregnancy without specifying gestational age—this was an amendment to the review protocol. Second trimester miscarriages without a clear definition were included only when the study presented stillbirths and/or foetal losses separately from miscarriages. Miscarriage was stratified for gestational age (first trimester miscarriage up to 12 weeks of gestation and second trimester miscarriage above 12 weeks of gestation).

Secondary outcomes were EP, defined as any extra-uterine pregnancy, TOP, defined as an induced abortion, and recurrent miscarriage, defined as a spontaneous miscarriage after two previous spontaneous miscarriages. We evaluated EP for completeness as early pregnancy loss includes EP. We evaluated TOP/induced abortions to ensure induced abortions were distinguished from spontaneous abortions.

### Statistical analysis

For studies comparing dichotomous outcomes in pregnant women with and without SARS-CoV-2 infection, we calculated odds ratios (OR) and risk differences (RDs) with corresponding 95% CI and pooled the data using random effects meta-analysis. To estimate the rate of miscarriage, EP, and TOP, we pooled proportions with 95% CI using DerSimonian and Laird random effects meta-analysis after transforming the data using Freeman–Tukey double arcsin transformation. Statistical heterogeneity between studies was reported as *I*^2^ statistics, *I*^2^ > 50% representing substantial heterogeneity. The impact of heterogeneity on pooled results was evaluated by calculating predictive intervals. We used the Metan and Metaprop routine in STATA for the analyses (StataCorp, 2019, Stata Statistical Software: Release 16, College Station, TX, USA: StataCorp LLC).

We aimed to describe miscarriage before 20 weeks of gestation per registered first and second trimester pregnancy and stratified for time of gestation (before and after 12 weeks of gestation). The study specific and summarized effect measures were calculated using a random effect model. When available, recurrent pregnancy loss was summarized as prevalence and OR with 95% CI, as described above.

For the prevalence estimates, we performed subgroup analyses per year of publication (2020, 2021, and 2022), per study region according to geographic World Bank regions (https://www.worldbank.org/), and on the basis of the study size (below or at least 20) to evaluate small study bias. A generalized linear mixed model (GLMM) instead of Freeman–Tukey double arcsin transformation was used as sensitivity analysis for the primary outcomes and GLMM was applied when zero counts resulted in inconsistencies in the estimates.

### Patient and public involvement

There was no patient or public involvement in the design and reporting of the present review.

## Results

### Search results

**Figure 1.**
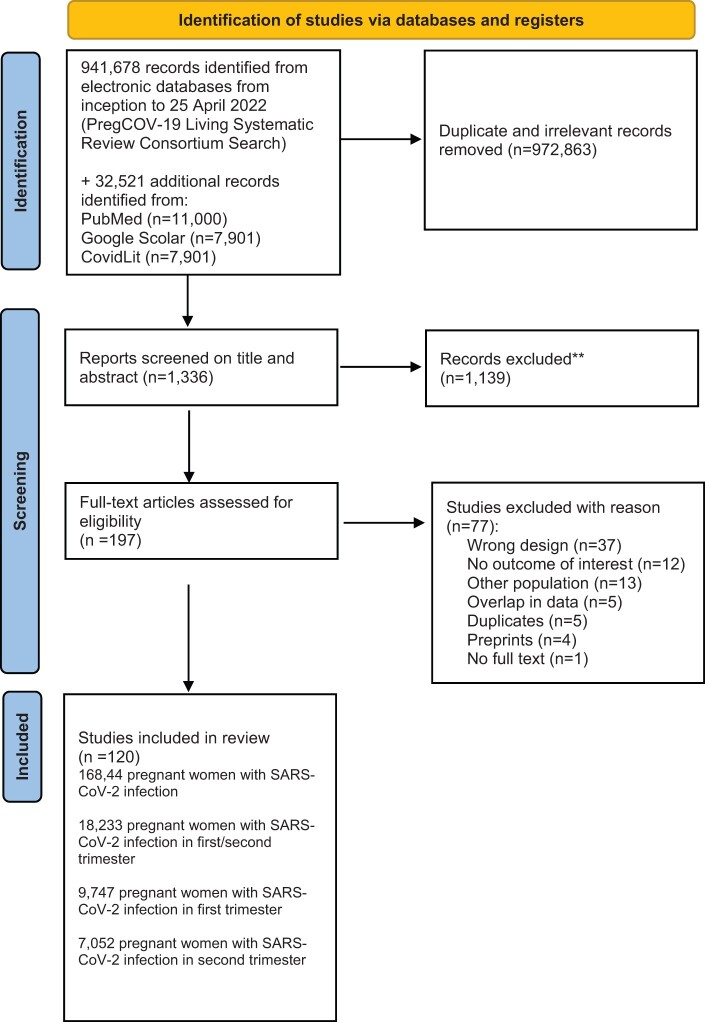
**PRISMA flowchart of selection of studies for a systematic review and meta-analysis on the risk and prevalence of pregnancy loss in pregnant women infected with SARS-CoV-2.** SARS-CoV-2, severe acute respiratory syndrome coronavirus 2.

A total of 941 678 citations were identified after screening electronic databases from inception to 25 April 2022 (PregCOV-19 Living Systematic Review Consortium search). Subsequently, with the additional electronic search from inception to 10 March 2023, conducted to specifically address pregnancy loss, we identified an additional 32 521 studies. The databases we used for the extra search were PubMed, Google Scholar, and the LitCovid Database. After the removal of duplicates and irrelevant articles, 1336 studies remained for screening; 1139 studies were excluded based on screening titles and abstracts. After screening the remaining 197 full text articles, we excluded 77 studies (Fig. 1).

Finally, we included 120 studies comprising a total of 168 444 pregnant women registered with SARS-CoV-2 infection in first or second trimester of pregnancy (Adhikari *et al*., 2020; Ayed *et al*., 2020; Calderón *et al*., 2020; Chen *et al*., 2020, 2021; Curi *et al*., 2020; Delahoy *et al*., 2020; Diouf *et al*., 2020; Edlow *et al*., 2020; Emeruwa *et al*., 2020; Fox and Melka, 2020; Grechukhina *et al*., 2020; Hernández *et al*., 2020, 2023; Kayem *et al*., 2020; Lei *et al*., 2020; London *et al*., 2020; Mattar *et al*., 2020; Metkari and Palve, 2020; Nambiar *et al*., 2020; Omrani *et al*., 2020; Pirjani *et al*., 2020; Qiancheng *et al*., 2020; Sentilhes *et al*., 2020; Shah *et al*., 2020; Shmakov *et al*., 2022; Tug *et al*., 2020; Woodworth *et al*., 2020; Wu *et al*., 2020a; Yan *et al*., 2020; Crovetto *et al*., 2021; Damar Çakırca *et al*., 2021, 2022; D’Antonio *et al*., 2021; Deng *et al*., 2021; Devi *et al*., 2021; Donati *et al*., 2021; Erol *et al*., 2021; Fan *et al*., 2021; Foo *et al*., 2021; Gajbhiye *et al*., 2021; Guo *et al*., 2021; Hazari *et al*., 2021; Hcini *et al*., 2021; Jacoby *et al*., 2021; Jang *et al*., 2021; Kuzan *et al*., 2021; Lokken *et al*., 2021; Mahajan *et al*., 2021; Manasova *et al*., 2021; Metz *et al*., 2021; Mullins *et al*., 2021; Overtoom *etal*., 2022; Poon *et al*., 2021; Priyadharshini *et al*., 2021; Aabakke *et al*., 2021, 2023; Abedzadeh-Kalahroudi *et al*., 2021; Ajith *et al*., 2021; Akram *et al*., 2021; Anand *et al*., 2021; Arinkan *et al*., 2021; Basu *et al*., 2021; Burwick *et al*., 2021; Cardona-Pérez *et al*., 2021; Chaudhary *et al*., 2021; Saimin *et al*., 2021; Santhosh *et al*., 2021; Singh *et al*., 2021; Taghavi *et al*., 2021; Tanacan *et al*., 2021; Vizheh *et al*., 2021; Vouga *et al*., 2021; Vousden *et al*., 2021; Yassa *et al*., 2021; Yazihan *et al*., 2021; Ahmad *et al*., 2022; Al-Hajjar *et al*., 2022; Arakaki *et al*., 2022; Babic *et al*., 2022; Balachandren *et al*., 2022; Barris *et al*., 2022; Bhoora *et al*., 2022; Borges-Charepe *et al*., 2022; Cambou *et al*., 2023; Chung *et al*., 2022; Collins *et al*., 2022; Cosma *et al*., 2022; Daclin *et al*., 2022; Fallach *et al*., 2022; Grandone *et al*., 2022; Hamadneh *et al*., 2022; Haye *et al*., 2022; Khoiwal *et al*., 2022; Kiremitli *et al*., 2022; Kumari *et al*., 2022; Martínez-Varea *et al*., 2022; McCreary *et al*., 2022; Neelam *et al*., 2023; Péju *et al*., 2022; Qudsieh *et al*., 2022; Regan *et al*., 2022; Rozo *et al*., 2022; Sahin *et al*., 2022a,b; Santos *et al*., 2022; Schell *et al*., 2022; Sekkarie *et al*., 2022; Souza *et al*., 2022; Sunder *et al*., 2022; Takemoto *et al*., 2022; Zelini *et al*., 2022; Ziert *et al*., 2022; Göklü *et al*., 2023; Hughes *et al*., 2023; Martinez-Baladejo *et al*., 2023; Poisson *et al*., 2023; Sertel and Demir, 2023; Tavakoli *et al*., 2023; Youssef *et al*., 2023). For studies with overlapping populations, we always selected the last most updated studies. In two cases both studies were included: Woodworth *et al*. (2020) had details on number of women in first and second trimester pregnancy that were lacking in Neelam *et al*. (2023); and Aabakke *et al*. (2021) had details on number of EP, but for all other outcomes we included the updated study (Aabakke *et al*., 2023).

Of the 120 included studies, 20 studies were cohort studies with a non-infected control group (Adhikari *et al*., 2020; Edlow *et al*., 2020; Pirjani *et al*., 2020; Cardona-Pérez *et al*., 2021; Crovetto *et al*., 2021; Donati *et al*., 2021; Erol *et al*., 2021; Gajbhiye *et al*., 2021; Jacoby *et al*., 2021; Metz *et al*., 2021; Taghavi *et al*., 2021; Tanacan *et al*., 2021; Yazihan *et al*., 2021; Balachandren *et al*., 2022; Cosma *et al*., 2022; Daclin *et al*., 2022; Fallach *et al*., 2022; Khoiwal *et al*., 2022; Souza *et al*., 2022; Sunder *et al*., 2022).

The details of the selection and review process are provided in Fig. 1.

### Risk of bias assessment of included studies

Supplementary Tables S2 and S3 illustrate the results of the risk of bias assessment. Overall, half of the prevalence studies were judged to be at moderate risk of bias (60/120), 39% were judged to be at low risk of bias (47/120), and 13 studies were judged to be of high risk of bias (13/120). In addition, 39% of cohort studies were judged to be at high risk of bias (7/18), 28% were judged to be at low risk of bias (5/18), and 33% were judged to be at medium risk of bias (6/18).

The following domains were considered as low risk of bias for external validity: representative of national population for relevant variables (population), representative of target population (sampling frame), some form of random selection was used to select the sample (selection bias), and more than 75% response rate in individuals with and without the outcome (non-response bias). The following domains were considered as low risk of bias for internal validity: all data were collected directly from the subjects, an acceptable case definition was used, the study instrument that measured the parameter of interest showed to have reliability and validity, the same mode of data collection was used for all subjects, the length of the shortest prevalence period of the parameter was appropriate and the paper provided appropriate numerators and denominators for the parameter of interest (Hoy *et al*., 2012).

### Characteristics of included studies

The 120 included studies assessed 168 444 pregnant women with SARS-CoV-2 infection. Most studies were from the USA (23), Turkey (13), India (13), China (9), Iran (5), Italy (4), France (4), with five studies performed in multiple countries. The majority of the women diagnosed with SARS-CoV-2 were in their third trimester of pregnancy, with 18 233 women in their first or second trimester of pregnancy.

Most studies confirmed diagnosis on the basis of SARS-CoV-2 RT-PCR (105/120); 15 studies tested for SARS-CoV-2 using either RT-PCR or antibodies to confirm the presence of SARS-CoV-2; one study confirmed a COVID-19 disease diagnosis through reports in the official COVID-19 test surveillance system of Brazil (Takemoto *et al*., 2022) and one study confirmed diagnosis based on clinical signs (Curi *et al*., 2020).

The average age ranged from 23 to 36 years in women with SARS-CoV-2 infection and from 27 to 34 years in the control group without SARS-CoV-2 infection. One study specifically reported on an infertile population that conceived following fertility treatment (Cousins, 2020).

The average BMI ranged from 23 to 33 kg/m^2^ in women with SARS-CoV-2 infection. Smoking was reported in 23 studies (Delahoy *et al*., 2020; Grechukhina *et al*., 2020; Kayem *et al*., 2020; WAPM, 2021; Aabakke *et al*., 2021; Cardona-Pérez *et al*., 2021; Crovetto *et al*., 2021; D’Antonio *et al*., 2021; Jacoby *et al*., 2021; Metz *et al*., 2021; Overtoom *etal*., 2022; Vouga *et al*., 2021; Vousden *et al*., 2021; Balachandren *et al*., 2022; Cosma *et al*., 2022; Daclin *et al*., 2022; Göklü *et al*., 2023; Hamadneh *et al*., 2022; Martínez-Varea *et al*., 2022; Hughes *et al*., 2023; Poisson *et al*., 2023; Souza *et al*., 2022; Martinez-Baladejo *et al*., 2023). One study reported on smoking marijuana (Edlow *et al*., 2020). Moreover, previous pregnancy loss was reported in two studies (Cosma *et al*., 2022; Sahin *et al*., 2022b). Characteristics of included studies are described in Supplementary Table S4.

The definition of miscarriage differed between studies and is reported in Supplementary Table S5. Seven studies defined miscarriage as pregnancy loss up to 24 weeks (Calderón *et al*., 2020; Emeruwa *et al*., 2020; Wu *et al*., 2020a; Akram *et al*., 2021; Poisson *et al*., 2023; Regan *et al*., 2022; Sertel and Demir, 2023).

One study provided mean gestational age for pregnancy loss in the second trimester (Schell *et al*., 2022). Forty-six studies did not provide a definition of miscarriage (Adhikari *et al*., 2020; Chen *et al*., 2020; Curi *et al*., 2020; Edlow *et al*., 2020; Emeruwa *et al*., 2020; Hernández *et al*., 2020; Sentilhes *et al*., 2020; Martinez-Baladejo *et al*., 2023; Metkari and Palve, 2020; Omrani *et al*., 2020; Pirjani *et al*., 2020; Ajith *et al*., 2021; Anand *et al*., 2021; Abedzadeh-Kalahroudi *et al*., 2021; Basu *et al*., 2021; Cardona-Pérez *et al*., 2021; Chaudhary *et al*., 2021; Crovetto *et al*., 2021; Donati *et al*., 2021; Guo *et al*., 2021; Jang *et al*., 2021; Saimin *et al*., 2021; Mahajan *et al*., 2021; Mullins *et al*., 2021; Overtoom *etal*., 2022; Poon *et al*., 2021; Priyadharshini *et al*., 2021; Tanacan *et al*., 2021; Yazihan *et al*., 2021; Vizheh *et al*., 2021; Vousden *et al*., 2021; Ahmad *et al*., 2022; Arakaki *et al*., 2022; Babic *et al*., 2022; Chung *et al*., 2022; Cosma *et al*., 2022; Daclin *et al*., 2022; Hamadneh *et al*., 2022; Sahin *et al*., 2022a; Souza *et al*., 2022; Zelini *et al*., 2022; Qudsieh *et al*., 2022; Regan *et al*., 2022; Santos *et al*., 2022; Ziert *et al*., 2022; Youssef *et al*., 2023).

### Miscarriage in women with SARS-CoV-2 infection versus non-infected controls

Eleven studies reported on miscarriage in the first or second trimester of pregnancy and had data on number of women in their first or second trimester of pregnancy with SARS-CoV-2 infection versus non-infected pregnant controls (Edlow *et al*., 2020; Crovetto *et al*., 2021; Donati *et al*., 2021; Erol *et al*., 2021; Taghavi *et al*., 2021; Tanacan *et al*., 2021; Yazihan *et al*., 2021; Balachandren *et al*., 2022; Cosma *et al*., 2022; Fallach *et al*., 2022; Souza *et al*., 2022). In the women with SARS-CoV-2 infection the average age was 30.4 (SD 3.0) years, and the average BMI was 25.3 kg/m^2^ (SD 4.7) versus 27.3 (SD 3.5) years and 25.0 kg/m^2^ (SD 5.1), respectively, in non-infected controls. Two studies were excluded from the meta-analysis: one study because presence of SARS-CoV-2 infection was based on self-reporting, which was an exclusion criterium (Balachandren *et al*., 2022), and the other study because miscarriage was only mentioned in the flowchart with unclear data in the control group (Donati *et al*., 2021).

Of the nine included studies, four studies were judged to have a low risk of bias (Crovetto *et al*., 2021; Tanacan *et al*., 2021; Yazihan *et al*., 2021; Fallach *et al*., 2022), two studies had a moderate risk of bias (Taghavi *et al*., 2021; Souza *et al*., 2022), and three a high risk of bias (Edlow *et al*., 2020; Erol *et al*., 2021; Cosma *et al*., 2022) (Supplementary Table S2).

**Figure 2.**
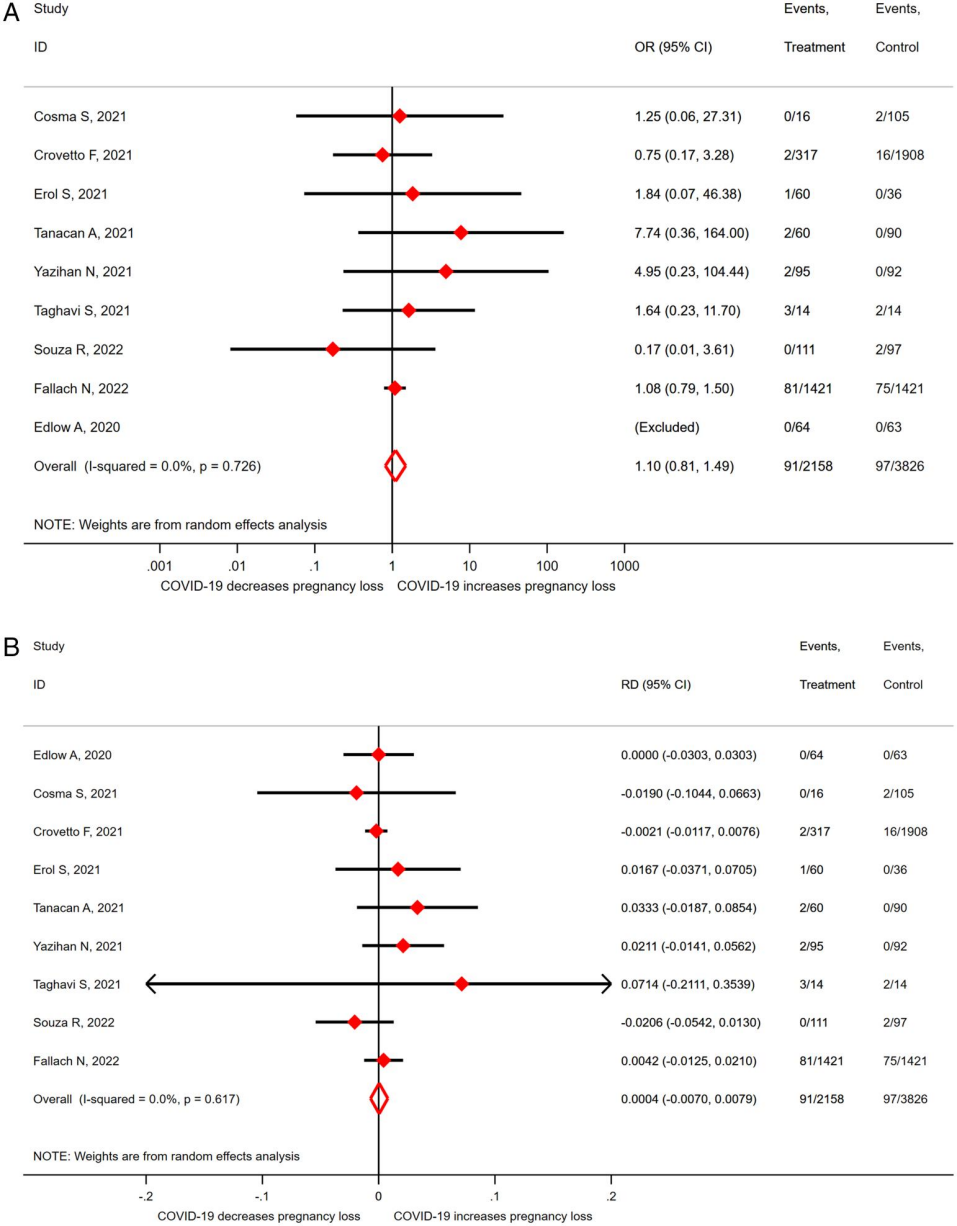
**Odds ratio (A) and risk difference (B) for miscarriage in pregnant women with SARS-CoV-2 versus without-SARS-CoV-2 infection.** SARS-CoV-2, severe acute respiratory syndrome coronavirus 2; COVID-19, coronavirus disease 2019; OR, odds ratio; RD, risk difference.

The OR for miscarriage in SARS-CoV-2 infected women versus non-infected controls was 1.10 (95% CI 0.81–1.49; *I*^2^ = 0.0%; 9 studies, 5984 women; moderate quality evidence; Fig. 2a). This corresponds to an RD of 0.0004 (95% CI −0.0070 to 0.0079; *I*^2^ = 0.0%; 9 studies, 5984 women; Fig. 2b). No stratified analyses were performed in view of the limited number of studies and lack of heterogeneity (*I*^2^ = 0). The majority of women were likely at the end of the first trimester; given a miscarriage rate of 10% in uninfected controls, the risk of a miscarriage in women with SARS-CoV-2 infection is between 9.3% and 10.8%.

### Proportion of first trimester miscarriage in women with SARS-CoV-2 infection

Miscarriage occurring in the first trimester was reported by 46 studies that included 1797 women diagnosed with SARS-CoV-2 infection in the first trimester (Curi *et al*., 2020; Edlow *et al*., 2020; Erol *et al*., 2021; Hernández *et al*., 2020, 2023; Grechukhina *et al*., 2020; Mattar *et al*., 2020; Metkari and Palve, 2020; Qiancheng *et al*., 2020; Shah *et al*., 2020; Tug *et al*., 2020; Wu *et al*., 2020a; Yan *et al*., 2020; Arinkan *et al*., 2021; Chen *et al*., 2021; Deng *et al*., 2021; ESHRE Working Group, 2021; Fan *et al*., 2021; Hazari *et al*., 2021; Guo *et al*., 2021; Jacoby *et al*., 2021; Jang *et al*., 2021; Kuzan *et al*., 2021; Lokken *et al*., 2021; Manasova *et al*., 2021; Santhosh *et al*., 2021; Singh *et al*., 2021; Yassa *et al*., 2021; WAPM, 2021; Al-Hajjar *et al*., 2022; Balachandren *et al*., 2022; Barris *et al*., 2022; Bhoora *et al*., 2022; Borges-Charepe *et al*., 2022; Cosma *et al*., 2022; Fallach *et al*., 2022; Khoiwal *et al*., 2022; Kiremitli *et al*., 2022; Kumari *et al*., 2022; McCreary *et al*., 2022; Sahin *et al*., 2022b; Santos *et al*., 2022; Schell *et al*., 2022; Shmakov *et al*., 2022; Souza *et al*., 2022; Sunder *et al*., 2022).

**Figure 3.**
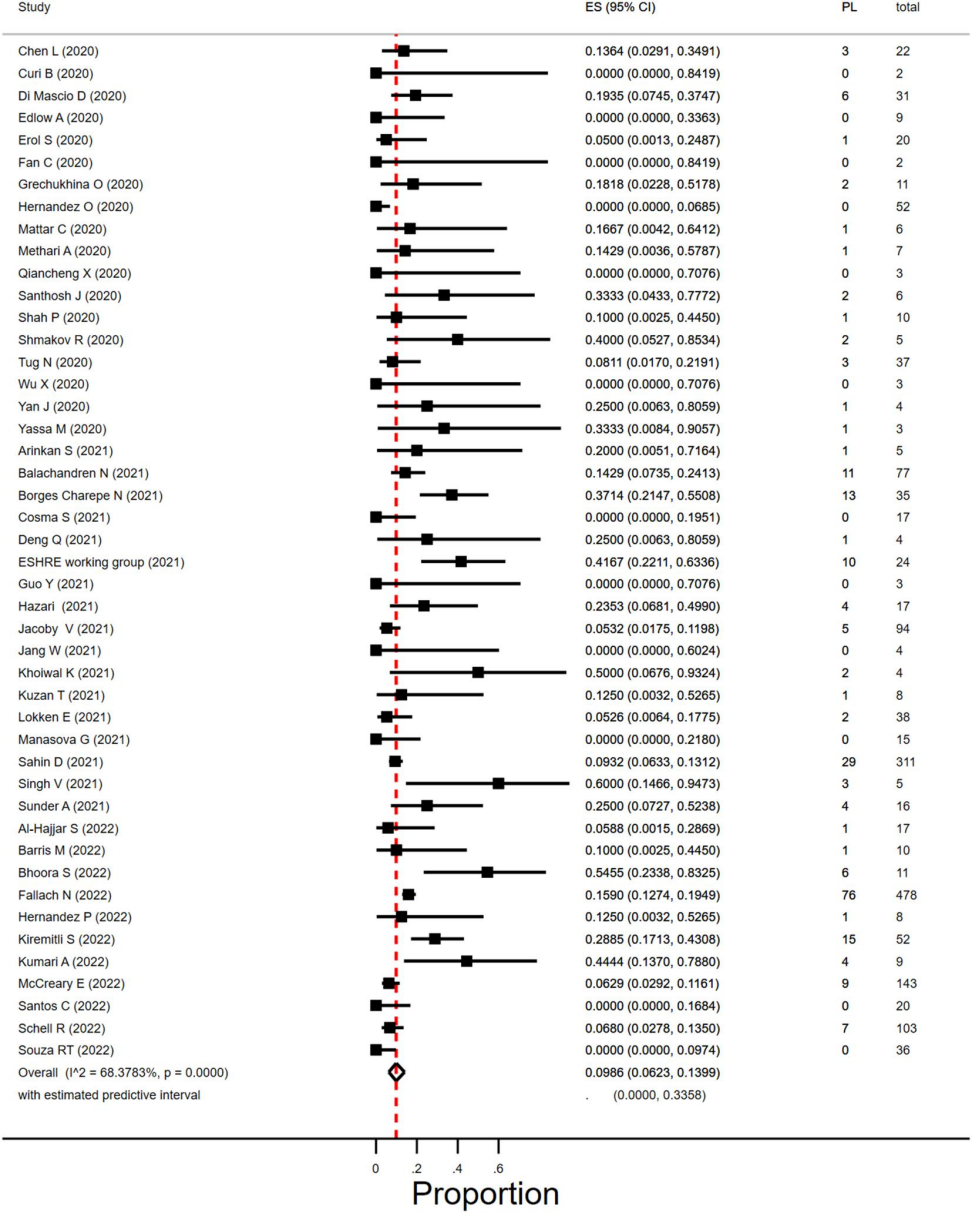
**Proportion of miscarriage in pregnant women with SARS-CoV-2 in the first trimester.** SARS-CoV-2, severe acute respiratory syndrome coronavirus 2; ES, estimate of proportion; PL, pregnancy loss.

In women with SARS-CoV-2 infection in their first trimester, the miscarriage rate was 9.9% (95% CI 6.2–14.0%; *I*^2^ = 68.4%; 46 studies, 1797 women; Fig. 3). There was substantial heterogeneity in the reported miscarriage estimates. The heterogeneity could partly be explained by differences in geographical region; in five studies from South Asia with 10 or less women in their first trimester of pregnancy, the miscarriage prevalence was between 10% and 60%. Subgroup analyses according to study size and year of publication resulted in overlapping estimates. Table 1 reports on overall, sensitivity, and subgroup results.

**Table 1.** Sensitivity and subgroup analyses for miscarriage in pregnant women with SARS-CoV-2 infection.

	Study N	Prevalence (95% CI)	I^2^	Subgroup heterogeneity
**First trimester miscarriage**	**46**	**9.86% (6.23–13.99)**	**68.4%**	
Sensitivity analysis with GLMM		10.02% (6.71–14.51)		
Sensitivity analysis fixed effect		7.85% (6.32–9.50)		
Sensitivity analysis with CC (0.5)		10.77% (7.69–13.85)		
Predictive interval		0–33.6%		
By World Bank region				***P* = 0.0003**
North America	8	6.24% (3.61–8.87)*	0.0%	
East Asia and Pacific	9	7.13% (0.02–19.27)	0.0%	
Europe and West Asia	13	13.98% (6.71–22.83)	75.6%	
Middle East and North Africa	5	14.70% (10.25–19.67)	13.6%	
South Asia	5	30.58% (11.70–52.66)	38.4%	
World, other regions	6	6.90% (0.00–22.83)	85.4%	
By study size, no of patients				*P* = 0.23
<20	33	10.93 (4.91–16.94)	50.0%	
≥20	13	9.73 (5.07–14.39)	74.1%	
By publication year				*P* = 0.80
2020	18	6.86 (1.56–14.30)	40.7%	
2021	17	12.81 (6.24–20.76)	71.8%	
2022	11	11.01 (4.84–18.83)	82.0%	
**Second trimester miscarriage**	33	1.24% (0.38–2.42)	58.8%	
Sensitivity analysis with GLMM		1.16% (0.73–2.43)		
Sensitivity analysis fixed effect		0.57% (0.28–0.87)		
Sensitivity analysis with CC (0.5)		1.58% (0.73–2.43)		
Predictive interval		0–3.8%		
By WB region				*P* = 0.24
North America	7	0.69% (0.00–1.99)	69.9%	
East Asia and Pacific	5	0.00% (0.00–5.22)	0%	
Europe and West Asia	8	1.12% (0.20–2.84)	0%	
Middle East and North Africa	4	8.75% (0.00–29.98)	72.5%	
Latin-America and Caribbean	4	0.77% (0.00–4.08)	67.2%	
South Asia	4	3.83% (0.00–22.17)	54.6%	
World, other regions	1	3.23% (0.08–16.70)		
By study size, no of patients				***P* = 0.004**
<20	18	2.91 (0.00–8.60)	31.3%	
≥20	15	1.13 (0.18–2.60)	78.1%	
By publication year				*P* = 0.09
2020	10	2.87 (0.00–10.95)	37.0%	
2021	11	1.13 (0.00–3.68)	40.7%	
2022	12	0.45 (0.00–1.96)*	46.7%	

* Unstable proportion owing to incorrect back transformation, recalculated with GLMM.

SARS-CoV-2, severe acute respiratory syndrome coronavirus 2; GLMM, generalized linear mixed models; CC, continuity correction.

### Proportion of second trimester miscarriage in women with SARS-CoV-2 infection

In 33 studies, the proportion of second trimester miscarriage could be retrieved (Adhikari *et al*., 2020; Curi *et al*., 2020; Grechukhina *et al*., 2020; Lei *et al*., 2020; Pirjani *et al*., 2020; Shah *et al*., 2020; Santhosh *et al*., 2021; Yan *et al*., 2020; Arinkan *et al*., 2021; Deng *et al*., 2021; ESHRE Working Group, 2021; Guo *et al*., 2021; Kuzan *et al*., 2021; Lokken *et al*., 2021; Manasova *et al*., 2021; Sunder *et al*., 2022; Yassa *et al*., 2021; Al-Hajjar *et al*., 2022; Barris *et al*., 2022; Bhoora *et al*., 2022; Borges-Charepe *et al*., 2022; Fallach *et al*., 2022; Khoiwal *et al*., 2022; Kumari *et al*., 2022; McCreary *et al*., 2022; Rozo *et al*., 2022; Sahin *et al*., 2022b; Santos *et al*., 2022; Schell *et al*., 2022; Shmakov *et al*., 2022; Souza *et al*., 2022; Hernández *et al*., 2023; Martinez-Baladejo *et al*., 2023). Second trimester miscarriage was usually defined as loss before 20 or 22 weeks. Two studies that did not provide a definition were also included as number of women in their second trimester and number of miscarriages during second trimester were provided (Curi *et al*., 2020; Souza *et al*., 2022).

**Figure 4.**
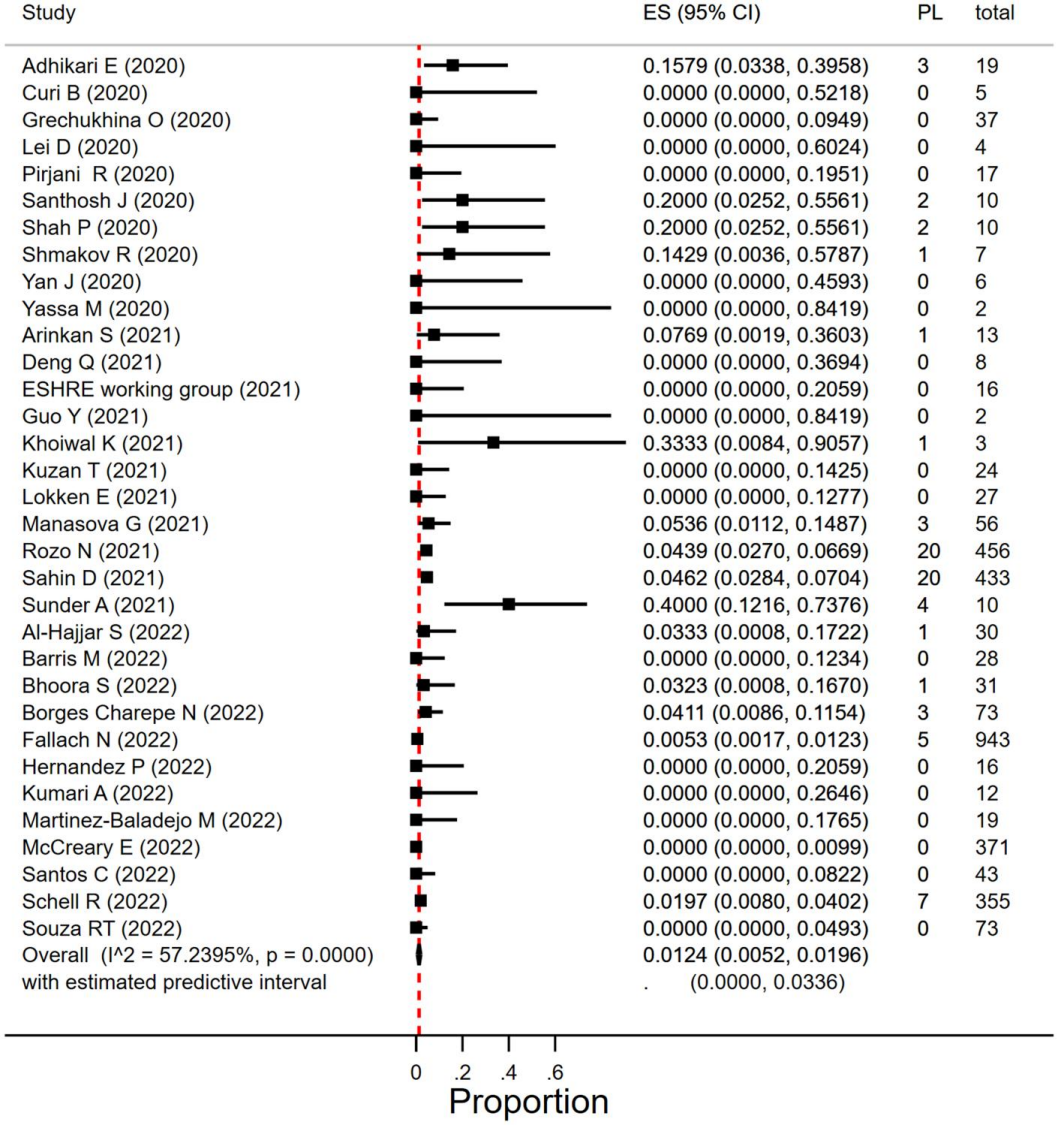
**Proportion of miscarriage in pregnant women with SARS-CoV-2 infection in the second trimester.** SARS-CoV-2, severe acute respiratory syndrome coronavirus 2; ES, estimate of proportion; PL, pregnancy loss.

The miscarriage rate in women with SARS-CoV-2 infection during the second trimester was 1.2% (95% CI 0.5–2.0%; *I*^2^ = 57%; 33 studies; 3159 women; Fig. 4 and Table 1). There was moderate heterogeneity in the reported miscarriage estimates between the studies. The proportion of second trimester miscarriages seemed to vary by size of the included study (*P* = 0.004) and was 2.9% (95% CI 0.0–8.6%) in studies that included <20 women and 1.1% (95% CI 0.2–2.6%) in studies with at least 20 women (Table 1). Subgroup analyses according to year of publication and geographical region resulted in overlapping estimates (respectively, *P* = 0.09 and *P* = 0.24) (Table 1).

### Additional analysis to estimate the prevalence of miscarriage in women with SARS-CoV-2 infection

In 42 studies, the number of miscarriages and number of women with SARS-CoV-2 infection and number of women in either first or second trimester was reported, but miscarriage rates could not be extracted for the first and second trimester separately. Supplementary Figure S1 provides an overall average for all studies that reported on miscarriage per number of women in either first or second trimester pregnancies. For the studies with incomplete trimester information the miscarriage rate in women that became infected at any time during their first or second trimester of pregnancy was 5.7% (95% CI 3.8–79%; *I*^2^ = 92%; 42 studies, 2909 women), and in the studies with trimester information 4.2% (95% 2.6–6.2%; *I*^2^ = 77%), leading to a total average of 4.9% (95% CI 3.6–6.4%; *I*^2^ = 88%; 92 studies). When assuming an equal distribution of women over the first and second trimester, the overall expected total chance to have a miscarriage would be twice these estimates; on basis of the calculated total average (95% CI of 3.61–6.44) this would be between 7.2% and 12.9%. This is in line with the sum of our reported first and second trimester estimates for miscarriage rate of 9.9% and 1.2%, respectively.

### Proportion of ectopic pregnancy in women with SARS-CoV-2 infection

We found 18 studies that reported on EP (Calderón *et al*., 2020; Chen *et al*., 2020; Curi *et al*., 2020; Metkari and Palve, 2020; Shah *et al*., 2020; Tug *et al*., 2020; Aabakke *et al*., 2021; Devi *et al*., 2021; Gajbhiye *et al*., 2021; Guo *et al*., 2021; Hazari *et al*., 2021; Singh *et al*., 2021; Balachandren *et al*., 2022; Bhoora *et al*., 2022; Khoiwal *et al*., 2022; Kumari *et al*., 2022; Martínez-Varea *et al*., 2022; Souza *et al*., 2022) but only for 15 studies could the number of women that were included in their first or second trimester of pregnancy be retrieved (Chen *et al*., 2020; Curi *et al*., 2020; Metkari and Palve, 2020; Shah *et al*., 2020; Tug *et al*., 2020; Aabakke *et al*., 2021; Guo *et al*., 2021; Hazari *et al*., 2021; Singh *et al*., 2021; Balachandren *et al*., 2022; Bhoora *et al*., 2022; Khoiwal *et al*., 2022; Kumari *et al*., 2022; Martínez-Varea *et al*., 2022; Souza *et al*., 2022). One study was excluded in view of an unexpectedly high number of EP suggesting extreme selection bias (four cases in seven women in their first trimester) (Metkari and Palve, 2020).

**Figure 5.**
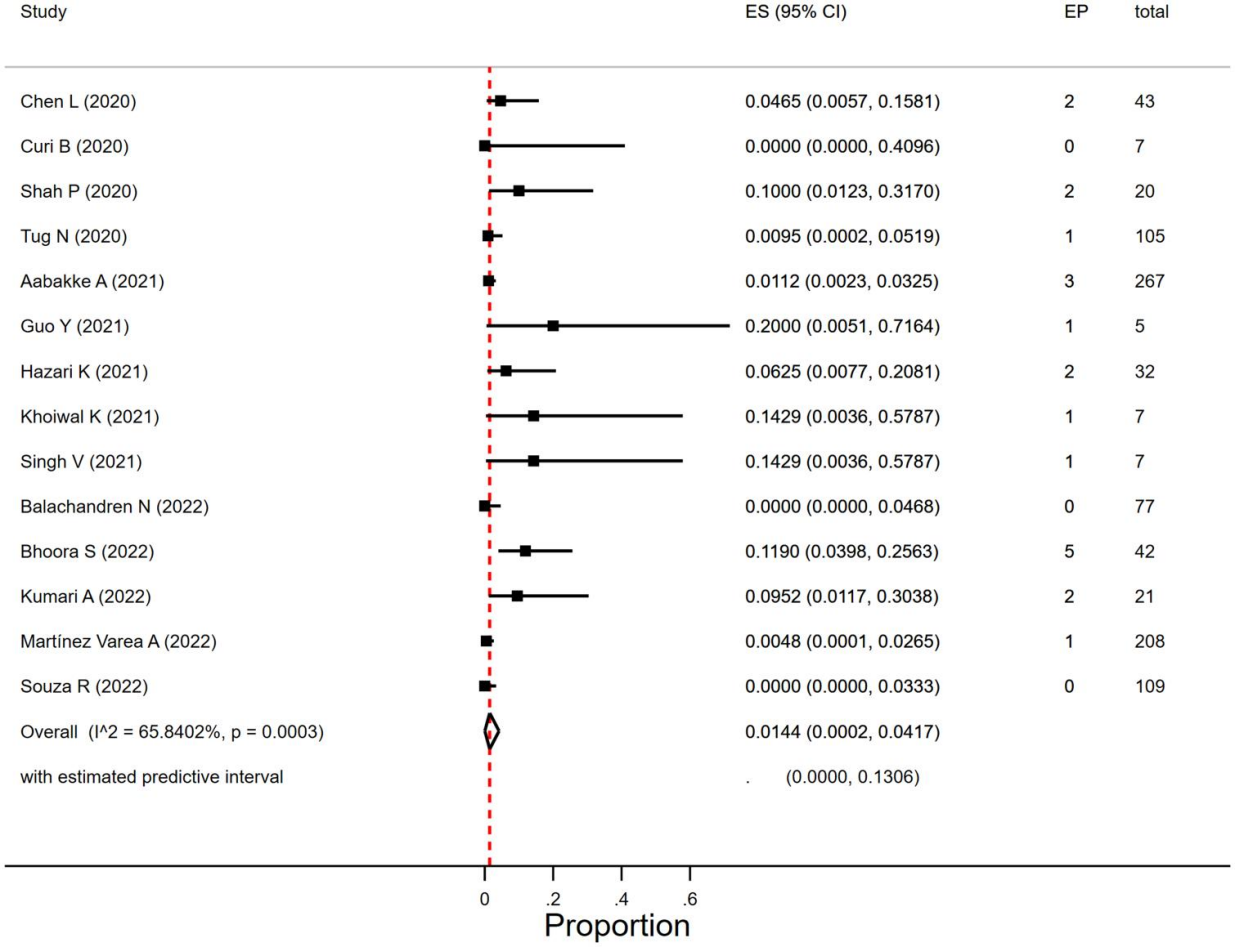
**Proportion of ectopic pregnancies in women with SARS-CoV-2 infection in the first or second trimester.** SARS-CoV-2, severe acute respiratory syndrome coronavirus 2; EP, ectopic pregnancy; ES, estimate of proportion.

The EP rate in women with SARS-CoV-2 infection was 1.4% (95% CI 0.02–4.2%; *I*^2^ = 65.8%; 14 studies, 950 women; Fig. 5). The heterogeneity was moderate to severe. In view of the limited number of studies we did not do subgroup analyses per geographical region on EP rate. The proportion of EP varied by size of study (*P* = 0.01) and was 1.0% (95% CI 0.0–3.4%) in 10 studies that included more than 20 women and 9.4% (95% CI 0.08–26.4) in studies with <20 women. Year of publication (2020, 2021, and 2022) did not affect EP rates (*P* = 0.41).

### Proportion of termination of pregnancy in women with SARS-CoV-2 infection

**Figure 6.**
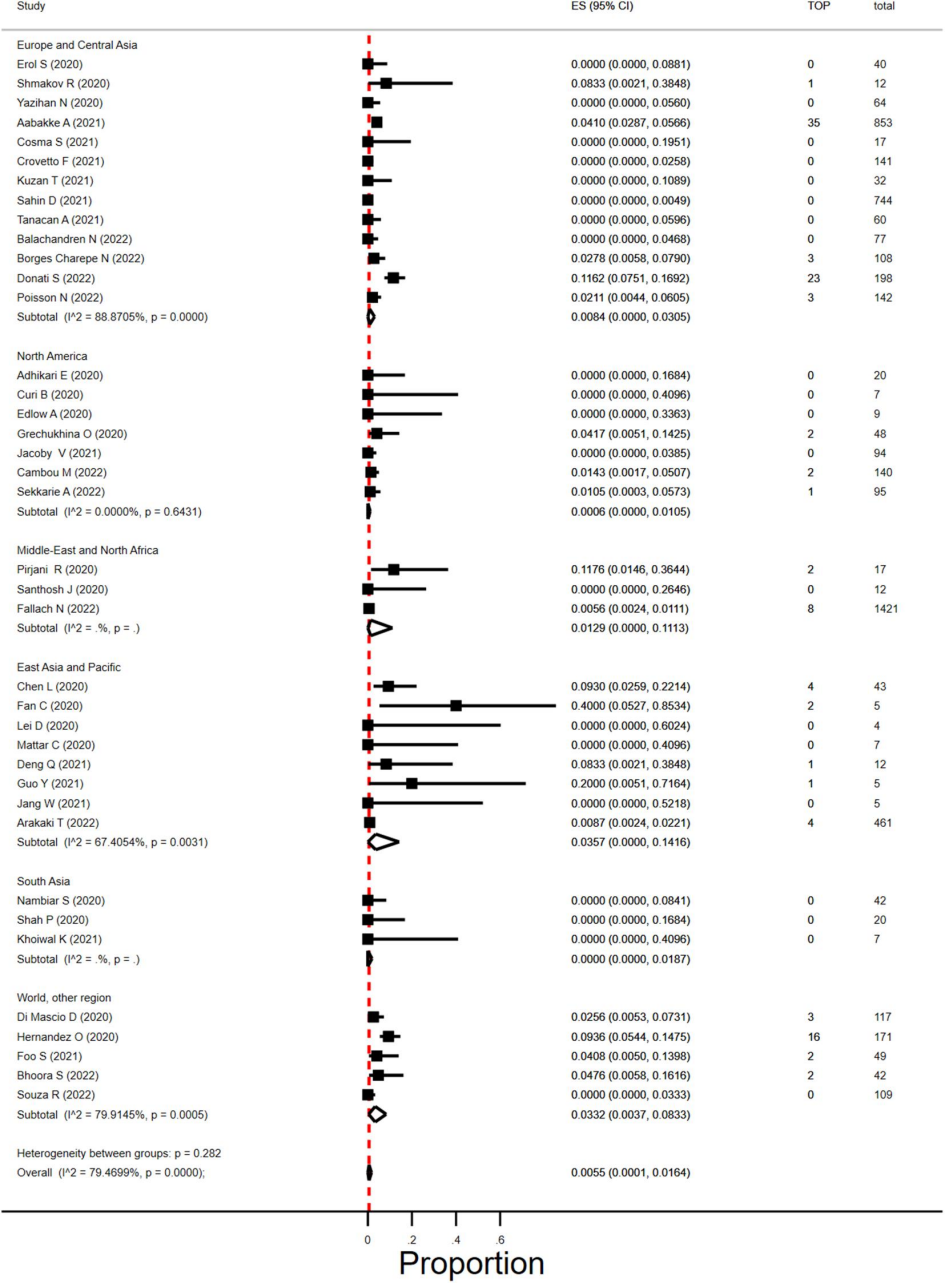
**Proportion of termination of pregnancies in women with SARS-CoV-2 infection in first or second trimester in different geographical regions.** SARS-CoV-2, severe acute respiratory syndrome coronavirus 2; TOP, termination of pregnancy; ES, estimate of proportion.

We found 49 studies that reported on TOP (Adhikari *et al*., 2020; Ayed *et al*., 2020; Chen *et al*., 2020; Curi *et al*., 2020; Edlow *et al*., 2020; Grechukhina *et al*., 2020; Hernández *et al*., 2020; Lei *et al*., 2020; London *et al*., 2020; Mattar *et al*., 2020; Nambiar *et al*., 2020; Pirjani *et al*., 2020; Qiancheng *et al*., 2020; Sentilhes *et al*., 2020; Shah *et al*., 2020; Wu *et al*., 2020a; Anand *et al*., 2021; Arinkan *et al*., 2021; Crovetto *et al*., 2021; Deng *et al*., 2021; Devi *et al*., 2021; Donati *et al*., 2021; Erol *et al*., 2021; Fan *et al*., 2021; Foo *et al*., 2021; Gajbhiye *et al*., 2021; Guo *et al*., 2021; Hcini *et al*., 2021; Jacoby *et al*., 2021; Jang *et al*., 2021; Kuzan *et al*., 2021; Mahajan *et al*., 2021; Santhosh *et al*., 2021; Tanacan *et al*., 2021; Vouga *et al*., 2021; Yazihan *et al*., 2021; Arakaki *et al*., 2022; Balachandren *et al*., 2022; Bhoora *et al*., 2022; Borges-Charepe *et al*., 2022; Cambou *et al*., 2023; Cosma *et al*., 2022; Fallach *et al*., 2022; Khoiwal *et al*., 2022; Poisson *et al*., 2023; Sahin *et al*., 2022b; Sekkarie *et al*., 2022; Shmakov *et al*., 2022; Souza *et al*., 2022; Aabakke *et al*., 2023) and, of these, 43 studies reported on the number of women in their first and/or second trimester of pregnancy (Adhikari *et al*., 2020; Ayed *et al*., 2020; Chen *et al*., 2020; Curi *et al*., 2020; Edlow *et al*., 2020; Grechukhina *et al*., 2020; Hernández *et al*., 2020; Lei *et al*., 2020; London *et al*., 2020; Mattar *et al*., 2020; Nambiar *et al*., 2020; Pirjani *et al*., 2020; Qiancheng *et al*., 2020; Sentilhes *et al*., 2020; Shah *et al*., 2020; Wu *et al*., 2020a; Crovetto *et al*., 2021; Deng *et al*., 2021; Donati *et al*., 2021; Erol *et al*., 2021; Fan *et al*., 2021; Foo *et al*., 2021; Guo *et al*., 2021; Jacoby *et al*., 2021; Jang *et al*., 2021; Kuzan *et al*., 2021; Santhosh *et al*., 2021; Tanacan *et al*., 2021; Yazihan *et al*., 2021; Arinkan *et al*., 2021; Arakaki *et al*., 2022; Balachandren *et al*., 2022; Bhoora *et al*., 2022; Borges-Charepe *et al*., 2022; Cambou *et al*., 2023; Cosma *et al*., 2022; Fallach *et al*., 2022; Khoiwal *et al*., 2022; Sahin *et al*., 2022b; Sekkarie *et al*., 2022; Shmakov *et al*., 2022; Souza *et al*., 2022; Aabakke *et al*., 2023; Poisson *et al*., 2023). Four studies were excluded, two studies as only one woman was in her first or second trimester of pregnancy (London *et al*., 2020; Sentilhes *et al*., 2020) and two studies as all of the women registered with an infection in their first or second trimester of pregnancies were TOP pregnancies (Qiancheng *et al*., 2020; Wu *et al*., 2020a). The overall proportion of termination in SARS-CoV-2 infected pregnancies was 0.6% (95% CI 0.01–1.6%; *I*^2^ = 79%; 39 studies; 1166 women; Fig. 6). Reason for TOP was only provided in a few early studies; in one early Chinese study two women asked for TOP owing to fear of SARS-CoV-2 effect on the pregnancy (Fan *et al*., 2021); in two studies TOP was medically indicated (Donati *et al*., 2021; Fan *et al*., 2021).

The proportion of TOP seemed to vary by size of the included study (*P* = 0.005) and was 2.5% (95% CI 0.01–7.6%) in studies that included <20 women and 1.3% (95% CI 0.39–2.5%) in studies with at least 20 women. Geographical regions did not differ in TOP rates (*P* = 0.28), nor did year of publication (2020, 2021, and 2022) (*P* = 0.28).

## Discussion

### Findings in context

In this systematic review, we found no evidence for an association of SARS-CoV-2 infection in early pregnancy with pregnancy loss. We found that pregnant women in the first or second trimester of pregnancy with SARS-CoV-2 infection were not at increased risk for a miscarriage compared to pregnant women without the infection. On average, first trimester miscarriage occurred in 1 in 10 pregnant women (10%) with SARS-CoV-2 infection and second trimester miscarriage in 12 of 1000 pregnant women (1.2%) with SARS-CoV-2 infection. These data suggest an overall miscarriage rate of 11%. This is in line with the estimate of the studies for which miscarriage rate per first and second trimester could not be extracted separately. These miscarriage rates also correspond to what would be expected in women without SARS-CoV-2 infection.

We found EP to occur in of 1.4% in early pregnancies. This compares to the overall rate of EP of 1–2% found in the general population (Panelli *et al*., 2015). The average proportion of TOP in women with SARS-CoV-2 infection was 0.6%.

Prevalence estimates showed substantial heterogeneity for first trimester miscarriage and moderate heterogeneity for second trimester miscarriage. For first trimester miscarriages, the prevalence differences between geographical region were largely caused by high prevalence in some small studies. Overall, study size and year of publication did not have significant effects on the estimates. Part of the heterogeneity in the first trimester could possibly be related to inclusion or exclusion of biochemical pregnancies, i.e. pregnancies diagnosed based on hCG. This would suggest that actual early miscarriage rates may be larger than estimated. On the other hand, in hospital-based studies selection bias is feasible, as women may be more inclined to visit the hospital in case of a miscarriage than when the pregnancy is ongoing, particularly when the pandemic was at the highest. For second trimester miscarriages the prevalence differed by study size and was highest in the smaller studies, with no significant effect of geographical region or year of publication. For EP heterogeneity was moderate, while it was substantial for TOP. TOP estimates varied by sample size of the individual study. Health care provider uncertainty of the effect of SARS-CoV-2 infection on mother and child may have increased the rate of TOP early in the pandemic (Wu *et al*., 2020b). On the other hand, lower abortion access because of COVID-19 pandemic-related restrictions may have resulted in a decrease in TOP (Cousins, 2020).

### How SARS-CoV-2 could be related to pregnancy loss

There is a known association of some viral infections with foetal malformation and pregnancy complications. Particular viruses can infect several cellular components of the placenta, such as trophoblasts, and can affect placental function, which may result in pregnancy complications such as preterm birth, miscarriage, and intrauterine growth restriction (Racicot and Mor, 2017). Also, it has been suggested that the adverse pregnancy outcomes following SARS-CoV-2 infection might be caused by an inflammatory cytokine imbalance (Vesce *et al*., 2022).

A review on 11 pregnant women infected with Middle East Respiratory Syndrome coronavirus (MERS-CoV) reported adverse outcomes in over 90% of the presented cases; there was no information related to placental infection (Alfaraj *et al*., 2019).

A study investigating the pregnancy and perinatal outcomes of pregnant women with SARS in 2002 reported that 57% of the patients had a miscarriage, while case fatality rate was 25%. Placental tissues and cord blood were negative for SARS-CoV in this study (Wong *et al*., 2004).

Viruses can directly infect the foetus at specific times during gestation. This can result in severe birth defects or even pregnancy loss (Racicot and Mor, 2017; Badr *et al*., 2020). How viruses cross the placental barrier and reach the foetus remains largely unknown. A proposed mechanism involves infection of extravillous trophoblasts and/or direct infection of maternal immune cells. Other possible routes of vertical transmission include direct infection of the syncytium or via inflammation-mediated disruption of the syncytiotrophoblast layer, thus damaging the placental barrier and allowing for transmission (Megli and Coyne, 2022). Results from a systematic review on mother-to-child transmission in SARS-CoV-2 infection confirmed vertical transmission of the virus could occur, although the absolute numbers were low and transmission was rare (Allotey *et al*., 2020).

Several case studies have reported on SARS-CoV-2 positive placental tissue in the second trimester using a variety of assays before 20 weeks of gestation (Baud *et al*., 2020; Michel *et al*., 2021). SARS-CoV-2-related placentitis, which is an inflammation of the placenta caused by infection with SARS-CoV-2 and is characterized by increased perivillous fibrin deposition, histiocytic intervillositis, and villous trophoblast necrosis (Mithal *et al*., 2022), has been associated with pregnancy loss and stillbirth (Stenton *et al*., 2022). On the other hand, another study found no placental differences between SARS-CoV-2 infected and non-infected women and suggested that maternal SARS-CoV-2-related respiratory failure and the resulting hypoxia is the major risk factor for pregnancy loss and stillbirth (Suhren *et al*., 2022). In addition, SARS-CoV-2 infection can trigger a cytokine storm, which may lead to both an inflammatory response in the foetus and to placental damage with consecutive foetal growth retardation, preterm birth, and miscarriage (Cavalcante *et al*., 2021).

The MaterCov study investigated the impact of SARS-CoV-2 infection on subclinical placental thrombosis and maternal thrombotic factors. They found an increased risk of developing obstetric complications in pregnant women with SARS-CoV-2, such as intrauterine growth restriction and stillbirth. However, there were no more placental pathologies identified in pregnant women infected by SARS-CoV-2 compared to pregnant women without infection (Carbonnel *et al*., 2022).

Given that SARS-CoV-2 infection during pregnancy is associated with more severe maternal disease than in SARS-CoV-2-infected non-pregnant women of reproductive age as well as associated with an increase in adverse perinatal outcomes such as stillbirth and preterm delivery with infection in later pregnancy, pregnant women should be advised to take precautions to avoid risk of SARS-CoV-2 exposure and receive a COVID-19 vaccine to reduce the risk of severe disease.

There is no reason to speculate that SARS-CoV-2 should be associated with abnormal fertilization and implantation outside the uterus. One study reported that SARS-CoV-2 can infect human embryo*s in vitro* (Montano *et al*., 2022); it remains to be elucidated whether this finding has any implications *in vivo*.

### Comparison with existing data

A recent European study (Balachandren *et al*., 2022) suggested that women who had SARS-CoV-2 infection in the first trimester had a higher risk of early miscarriage. The authors found an early miscarriage rate of 14% in the ‘presumed infected’ group (11/77 [95% CI 6–22]), 5% in the ‘uncertain’ group (15/295 [95% CI 3–8]), and 8% in the ‘presumed uninfected’ group (212/2669 [95% CI 7–9], *P* = 0.02). After adjusting for age, BMI, ethnicity, smoking status, gestational age at registration and the number of previous miscarriages, they found the risk of early miscarriage to be higher among women with presumed SARS-CoV-2 infection in the first trimester compared to those with no infection (relative rate 1.7, 95% CI 1.0–3.0, *P* = 0.06). This study used self-reported data on diagnosis of SARS-CoV-2 and pregnancy outcomes, which may have resulted in higher reporting of miscarriage in the presumed infected group.

Two systematic reviews and meta-analyses reported on the proportion of miscarriage in women with SARS-CoV-2. One review included seven cases-series and concluded higher miscarriage rates were found in infected women (Kazemi *et al*., 2021), the other review included 17 studies, both case-series and cohort studies, and suggested miscarriage rates were comparable to non-infected women (Cavalcante *et al*., 2021). A recent systematic review and meta-analysis reported, among several other outcomes, that there was no significant difference in the rates of total miscarriage between SARS-CoV-2-infected and non-infected pregnant women (Jeong and Kim, 2023). In this review, the denominator included women in their third trimester both in the infected and non-infected controls, thus not allowing a fair comparison.

A systematic review on foetal demise, including stillbirths and late miscarriages following SARS-CoV-2 infection, concluded that most cases with late miscarriages (between 14 and 22 weeks) and stillbirths presented with placental abnormalities associated with potential transplacental SARS-CoV-2 infection, which may cause placental insufficiency and foetal hypoxia. The review included mostly case studies and case series (Alcover *et al*., 2023).

### Study strengths and limitations

The strength of this systematic review and meta-analysis is the review process that combined the extensive search of the PregCov consortium with specific searches, which enabled us to include a large population of pregnant women with and without SARS-CoV-2 infection during the first and second trimester.

Our review has several limitations. Most included studies were hospital-based studies such that selection bias towards more severe infections seems likely. On the other hand, if the prevalence of miscarriage is not clearly higher in the women with more severe COVID-19, then it is very unlikely it will be higher in infection with mild symptoms. The majority of studies did not include very early pregnancies, i.e. before 5 or 6 weeks of gestation. This implies that we cannot make a statement on the possible effect of SARS-CoV-2 infection on biochemical pregnancy. However, as the majority of biochemical pregnancies miscarry owing to chromosomal abnormalities, and as comparable viral infections, such as influenza, only increased miscarriage rates later in pregnancy, it does not seem likely that SARS-CoV-2 is associated with very early miscarriage (Dawood *et al*., 2021). The effect estimates have large 95% confidence boundaries and there was high heterogeneity across studies in first trimester miscarriage. This could only partly be explained by small study effect and may further be explained by differences in definition of miscarriage and differences in gestational age at which women were included in the cohorts and registries. Therefore, uncertainties remain for key outcomes that require further evidence. Furthermore, different variants of SARS-CoV-2, treatment of pregnant women with COVID-19 and access to vaccines could have affected outcomes but could not be studied as detailed data on variants, treatment and prevention in women in their first of second trimester of pregnancy was usually lacking. SARS-CoV-2 variants differed between 2020 and 2022, however, prevalence estimates for 2020, when no treatment was available yet, overlapped with prevalence estimates for 2021 and 2022. Meanwhile, a large proportion of vaccinated women could only be expected in the studies published in 2022. The overlapping estimates between 2020, 2021, and 2022 suggest there is no large impact of variants of SARS-CoV-2, treatment of pregnant women with COVID-19 and access to vaccines on pregnancy loss. Additionally, data on prior pregnancy history, such as previous pregnancy loss, were mostly not available. These could be important confounders when assessing the risk of miscarriages.

### Relevance for clinical practice and research

In order to provide better risk estimates more studies are needed. These are preferably well-designed prospective studies that include pregnant women with and without SARS-CoV-2 infection at conception and early pregnancy, and consider the association of clinical manifestation and severity of COVID-19 disease with pregnancy loss, as well as potential confounding factors such as previous pregnancy loss. In SARS-CoV-infected women with a miscarriage, foetal karyotyping could be carried out to exclude a genetic factor.

Even though the WHO has declared an end to the COVID-19 pandemic as a global health emergency, women are at risk of SARS-CoV-2 infection. Pregnant women should be advised to take precautions to avoid risk of SARS-CoV-2 exposure and to be vaccinated with a COVID-19 vaccine.

## Conclusion

There are still many unknowns regarding SARS-CoV-2 infection in early pregnancy. Reassuringly, based on currently available evidence, there are no indications that SARS-CoV-2 infection in early pregnancy increases the risk of miscarriages. In order to provide better risk estimates, well-designed studies are needed.

## Supplementary Material

dmad030_Supplementary_Data

## Data Availability

Data collected including data on pregnancy loss is available at https://cgf.cochrane.org/news/covid-19-coronavirus-disease-fertility-and-pregnancy. Dissemination to participants and related patient and public communities: The PregCov-19 LSR Group will disseminate the findings through dedicated websites: www.birmingham.ac.uk/research/who-collaborating-centre/pregcov/index.aspx and https://cgf.cochrane.org/news/covid-19-coronavirus-disease-fertility-and-pregnancy as well as through social media.
