# Supplementary material for: COVID-19 in pregnant women: a systematic review and meta-analysis on the risk and prevalence of pregnancy loss
Source: Hum Reprod Update. 2023 Nov 28;30(2):133–52. doi: 10.1093/humupd/dmad030 (PMC10905512; doi:10.1093/humupd/dmad030)

**Supplementary Table S1. Search terms in PubMed/Google Scholar/CovidLit**

| **Search terms PubMed** | |
| --- | --- |
| **1.** | abortion, spontaneous [MeSH Terms] |
| **2.** | miscarriage[tw] |
| **3.** | pregnancy loss[tw] |
| **4.** | abortion[tw] |
| **5.** | pregnancy, ectopic [MESH Terms] |
| **6.** | ectopic pregnancy[tw] |
| **7.** | pregnancy outcome [MeSH Terms] |
| **8.** | pregnancy outcome[tw] |
| **9.** | termination of pregnancy[tw] |
| **10.** | abortion, induced [MeSH Terms] |
| **11.** | Pregnancy [MESH Terms] |
| **12.** | pregnancy.tw |
| **13.** | infant, newborn [MeSH Terms] |
| **14.** | neonatal[tw] |
| **15.** | maternal.tw |
| **16.** | obstetric.tw |
| **17.** | 1-16, OR |
| **18.** | COVID-19[All fields] |
| **19.** | COVID-19[MeSH Terms] |
| **20.** | COVID-19[tw] |
| **21.** | SARS-CoV-2[MeSH Terms] |
| **22.** | SARS-CoV-2[tw] |
| **23.** | 18-23, OR |
| **24.** | 17 AND 24 |
|  |  |
| **Search terms Google Scholar/CovidLit** | |
|  | The following text words were used |
| **1** | Pregnancy loss OR miscarriage OR abortion OR termination of pregnancy |
| **2** | Ectopic pregnancy |
| **3** | Pregnancy |
| **4** | 1 OR 2 OR 3 |
| **5** | COVID-19 or SARS-CoV-2 |
| **6** | 1 AND 2 |

# **Supplementary Table S2. Risk of bias assessment of included cohort studies (Newcastle-Ottawa Scale)**


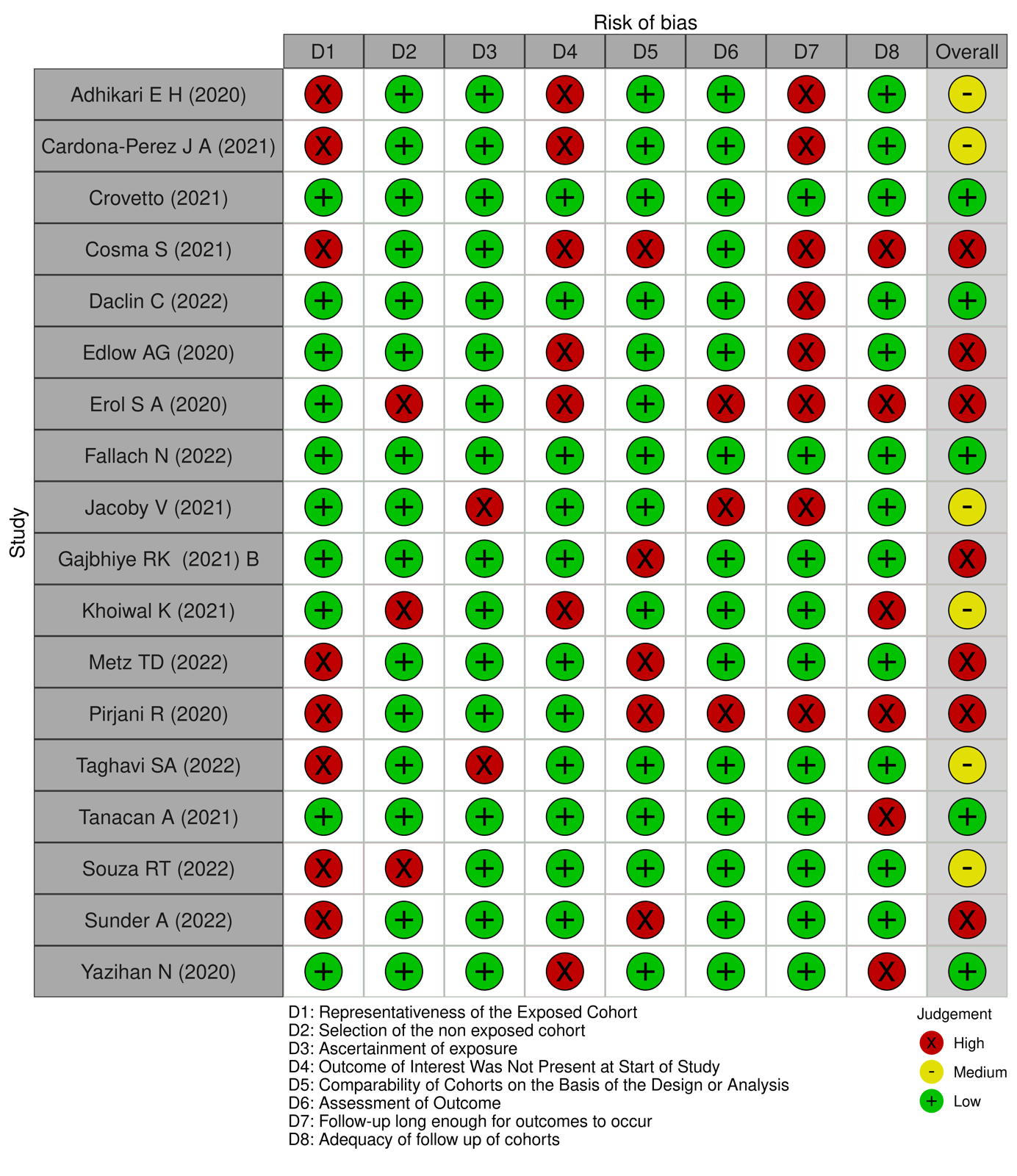


# **Supplementary Table S3. Risk of bias assessment of included prevalence studies (Hoy et al. )**

| **Study** | **Country** | **Represen-tativeness** | **Sampling frame** | **Selection** | **Non-response** | **Data collection** | **Case definition** | **Measure-ment** | **Differential verification** | **Adequate follow up** | **Appropriate numerator and denominator** | **Summary** |
| --- | --- | --- | --- | --- | --- | --- | --- | --- | --- | --- | --- | --- |
| Aabakke AJM 2023 | Denmark | Low | Low | High | Low | Low | Low | Low | Low | Low | Low | **Low** |
| Aabakke AJM 2021 | Iran | High | Low | High | Low | Low | High | Low | Low | High | Low | **Moderate** |
| Abedzadeh-Kalahroudi M  2021 | USA | High | Low | High | Low | Low | High | Low | Low | High | Low | **Moderate** |
| Adhikari EH 2020 | India | High | Low | High | Low | Low | High | Low | Low | High | Low | **Moderate** |
| Ahmad SN 2022 | Iraq | High | Low | High | Low | Low | High | Low | Low | High | Low | **Moderate** |
| Ajith S  2021 | India | High | Low | High | Low | Low | High | Low | Low | High | Low | **Moderate** |
| Akram EG  2021 | USA | Low | Low | High | Low | Low | High | Low | Low | High | Low | **Low** |
| Al-Hajjar S 2022 | Turkey | High | High | High | Low | Low | High | Low | Low | Low | Low | **Moderate** |
| Anand P 2021 | Kuwait | Low | Low | High | Low | Low | High | Low | Low | High | Low | **Low** |
| Arakaki T 2022 | UK | Low | Low | High | Low | Low | Low | Low | Low | High | Low | **Moderate** |
| Arinkan  SA 2021 | South Africa | Low | Low | High | Low | Low | High | Low | Low | Low | Low | **Low** |
| Ayed A  2020 | Portugal | High | High | High | Low | Low | High | Low | Low | High | Low | **Moderate** |
| Babic I  2022 | USA | Low | High | High | Low | Low | High | Low | Low | High | Low | **Moderate** |
| Balachandren N  2022 | Turkey | Low | High | High | Low | High | Low | Low | Low | Low | Low | **Low** |
| Barris M 2022 | Mexico | Low | High | High | Low | Low | High | Low | Low | High | Low | **Moderate** |
| Basu JK 2021 | Mexico | High | Low | High | Low | Low | High | Low | Low | High | Low | **Moderate** |
| Bhoora S 2022 | China | High | Low | High | Low | Low | High | Low | Low | High | Low | **Moderate** |
| Borges-Charepe N 2022 | USA | Low | Low | High | Low | Low | Low | Low | Low | High | Low | **Low** |
| Burwick RM 2021 | Spain | Low | Low | High | Low | Low | Low | Low | Low | High | Low | **Low** |
| Çakirca  TD 2021 | Italy | High | High | High | Low | High | High | High | Low | High | Low | **High** |
| Calderón JM  2020 | USA | High | High | High | Low | Low | High | Low | High | High | Low | **Moderate** |
| Cambou MC 2022 | France | High | High | High | Low | Low | High | Low | Low | High | Low | **Moderate** |
| Cardona-Pérez JA 2021 | USA | Low | Low | High | Low | Low | High | Low | Low | High | Low | **Low** |
| Chaudhary S 2021 | China | Low | Low | High | Low | Low | Low | Low | Low | High | Low | **Low** |
| Chen L 2020 | India | Low | High | High | Low | Low | High | Low | Low | High | Low | **Moderate** |
| Chen S  2021 | Worldwide | Low | High | Low | Low | Low | Low | Low | Low | High | High | **Low** |
| Chung Y 2022 | Senegal | High | High | High | Low | Low | Low | Low | Low | High | Low | **Moderate** |
| Cosma  2021 | Italy | Low | Low | High | High | Low | High | Low | Low | High | Low | **Moderate** |
| Crovetto  2021 | USA | High | Low | High | Low | Low | High | Low | Low | High | Low | **Moderate** |
| Curi B 2020 | USA | High | Low | Low | Low | Low | Low | Low | Low | High | Low | **Low** |
| Daclin  2022 | Turkey | Low | High | High | Low | Low | High | Low | Low | High | Low | **Moderate** |
| d'Antonio  2021 | Worldwide | High | Low | Low | High | Low | High | Low | Low | High | Low | **Moderate** |
| Delahoy  2020 | China | High | High | High | Low | Low | High | Low | Low | High | Low | **Moderate** |
| Deng Q 2021 | USA/Brazil | Low | Low | High | Low | Low | Low | Low | Low | High | Low | **Low** |
| Devi  2021 | USA | High | High | High | Low | Low | High | Low | Low | High | Low | **Moderate** |
| Di Mascio D 2021 | India | Low | Low | High | Low | Low | High | Low | Low | High | Low | **Low** |
| Diouf  2020 | Italy | High | High | High | Low | Low | Low | Low | Low | High | Low | **Moderate** |
| Donati  2021 | USA | High | Low | High | Low | Low | High | Low | Low | High | Low | **Moderate** |
| Edlow AG  2020 | China | High | Low | High | Low | Low | High | Low | Low | High | Low | **Moderate** |
| Emeruwa  2020 | Chile | High | Low | High | Low | Low | High | Low | Low | High | Low | **Moderate** |
| Erol SA 2021 | Dubai | Low | Low | High | High | Low | High | Low | Low | Low | Low | **Low** |
| ESHRE working group 2021 | French Guinea | Low | High | High | Low | Low | High | Low | Low | High | Low | **Moderate** |
| Fallach N 2022 | Chile | High | Low | High | Low | Low | High | Low | Low | High | Low | **Moderate** |
| Fan C 2021 | USA | High | Low | High | Low | High | High | Low | Low | High | Low | **Moderate** |
| Foo  2021 | Korea | High | High | High | Low | Low | High | Low | Low | High | Low | **High** |
| Fox  2020 | France | High | Low | High | Low | Low | High | Low | Low | High | Low | **Moderate** |
| Gajbhiye  2021 A | India | High | High | High | Low | Low | High | Low | Low | High | Low | **Moderate** |
| Göklü MR 2023 | Turkey | High | High | High | High | Low | High | Low | Low | High | Low | **Moderate** |
| Grandone  2022 | China | High | High | High | Low | Low | High | Low | Low | High | Low | **Moderate** |
| Grechukhina O 2020 | USA | Low | Low | High | Low | Low | Low | Low | Low | High | Low | **Low** |
| Guo Y 2021 | USA | High | Low | High | Low | Low | High | Low | Low | High | Low | **Moderate** |
| Hamadneh J 2022 | India | Low | Low | High | Low | High | Low | Low | Low | High | Low | **Low** |
| Haye  2021 | Ukraine | High | High | High | Low | Low | Low | Low | Low | High | Low | **Moderate** |
| Hazari KS 2021 | Singapore | High | High | High | Low | Low | High | Low | Low | Low | Low | **Moderate** |
| Hcini  2020 | USA | High | High | High | Low | Low | Low | Low | Low | High | Low | **Moderate** |
| Hernandez PV 2023 | PAN-COVID | Low | Low | High | Low | Low | High | Low | Low | High | Low | **Low** |
| Hernandez BO  2020 | India | High | Low | High | Low | Low | Low | Low | Low | High | Low | **Low** |
| Hughes BL 2023 | The Netherlands | Low | Low | High | Low | Low | High | Low | Low | High | Low | **Low** |
| Jacoby  2021 | Qatar | Low | High | Low | Low | Low | High | Low | Low | High | Low | **Low** |
| Jang WK 2021 | Hong Kong | High | High | High | High | Low | High | Low | Low | High | Low | **Moderate** |
| Kayem  2020 | Iran | High | High | High | Low | Low | High | Low | Low | High | Low | **Moderate** |
| Khoiwal K 2021 | India | Low | Low | High | Low | Low | Low | Low | High | High | Low | **Low** |
| Kiremitli S 2022 | China | High | Low | High | Low | Low | High | Low | Low | High | Low | **Moderate** |
| Kumari A 2022 | USA | High | High | High | Low | Low | High | Low | High | High | Low | **Moderate** |
| Kuzan TY 2021 | Colombia | High | High | High | Low | High | High | Low | High | High | Low | **High** |
| Lei D 2020 | Turkey | High | Low | High | Low | Low | Low | Low | Low | High | Low | **Low** |
| Lokken EM  2021 | Turkey | High | High | High | Low | Low | High | Low | Low | High | Low | **Moderate** |
| London  2020 | Indonesia | High | High | High | Low | Low | High | Low | High | High | Low | **High** |
| Mahajan NN 2021 | Oman | Low | Low | High | Low | Low | High | Low | High | High | Low | **Moderate** |
| Manasova G 2021 | France | Low | High | High | Low | Low | High | Low | Low | High | Low | **Moderate** |
| Martínez Varea A 2022 | India | Low | Low | High | Low | Low | Low | Low | Low | High | Low | **Low** |
| Martinez-Baladejo MT 2023 | Russia | Low | Low | High | Low | Low | High | Low | Low | High | Low | **Low** |
| Mattar CN 2020 | India | Low | High | High | Low | Low | High | Low | Low | High | Low | **Moderate** |
| McCreary EK 2022 | Bahrein | High | Low | High | Low | Low | Low | Low | Low | Low | Low | **Moderate** |
| Metkari AM 2020 | Brazil | Low | High | Low | Low | Low | High | Low | Low | High | Low | **Low** |
| Metz  2022 | Turkey | Low | High | High | Low | Low | High | Low | Low | High | Low | **Moderate** |
| Mullins E 2021 | Turkey | Low | High | High | Low | High | High | High | Low | High | Low | **High** |
| Nambiar SS 2020 | Iran | Low | High | High | Low | Low | High | Low | Low | High | Low | **Moderate** |
| Neelam V 2022 | Switzerland | High | High | High | Low | Low | High | High | Low | High | Low | **High** |
| Omrani AS 2020 | UK | High | High | High | Low | Low | Low | Low | High | High | Low | **Moderate** |
| Overtoom EM 2021 | World | Low | Low | High | Low | Low | High | Low | Low | High | Low | **Low** |
| Péju E 2022 | USA | Low | High | Low | Low | High | High | Low | Low | High | Low | **Moderate** |
| Pirjani R 2020 | China | High | High | High | Low | High | High | High | Low | High | Low | **High** |
| Poisson M 2023 | China | High | High | High | Low | Low | High | Low | Low | High | High | **Moderate** |
| Poon LC 2020 | Turkey | High | Low | Low | Low | Low | Low | Low | Low | High | Low | **Low** |
| Priyadharshini C 2021 | Turkey | Low | Low | High | Low | Low | High | Low | Low | High | Low | **Low** |
| Qiancheng X 2020 | Germany | High | Low | High | High | Low | High | Low | Low | Low | Low | **Moderate** |
| Qudsieh S 2022 | Turkey | Low | High | High | Low | Low | Low | Low | Low | Low | Low | **Low** |
| Regan A 2022 | India | Low | High | High | Low | Low | High | Low | Low | Low | Low | **Moderate** |
| Rozo N 2021 | Brazil | Low | Low | High | Low | High | High | Low | Low | Low | Low | **Low** |
| Sahin D 2021 | Israel | Low | Low | High | Low | Low | Low | Low | Low | Low | Low | **Low** |
| Sahin D 2022 | Saudi Arabia | High | High | High | Low | Low | High | Low | Low | Low | Low | **Moderate** |
| Saimin J 2021 | Italy | High | High | High | Low | Low | High | Low | Low | Low | Low | **Moderate** |
| Santhosh J 2020 | USA | High | Low | High | Low | Low | Low | Low | Low | Low | Low | **Low** |
| Santos CAD 2022 | USA | High | Low | High | Low | Low | High | Low | Low | Low | Low | **Low** |
| Schell RC 2022 | European | High | Low | High | Low | Low | Low | Low | Low | Low | Low | **Low** |
| Sekkarie A 2022 | USA | Low | Low | High | Low | Low | High | Low | Low | Low | Low | **Low** |
| Sentilhes L 2020 | USA | Low | Low | High | Low | Low | Low | Low | Low | Low | Low | **Low** |
| Sertel E 2023 | India | High | High | High | Low | Low | High | Low | Low | Low | Low | **Moderate** |
| Shah PT 2020 | USA | High | Low | High | Low | Low | Low | Low | Low | Low | Low | **Low** |
| Shmakov RG 2020 | USA | High | High | High | Low | Low | High | Low | Low | High | Low | **High** |
| Singh V 2021 | Brazil | High | High | High | Low | Low | High | Low | Low | Low | Low | **Moderate** |
| Souza RT 2022 | Jordan | High | High | High | Low | Low | High | Low | Low | High | Low | **High** |
| Sunder A 2022 | Korea | High | Low | High | Low | Low | High | Low | Low | Low | Low | **Low** |
| Taghavi SA 2021 | USA | High | High | High | Low | Low | Low | Low | Low | Low | Low | **Low** |
| Takemoto MLS 2020 | Saudi Arabia | High | High | High | Low | Low | Low | Low | Low | Low | Low | **Low** |
| Tanacan A 2021 | Turkey | High | High | High | Low | Low | Low | Low | Low | Low | Low | **Low** |
| Tavakoli N 2023 | Jordan | High | High | High | High | Low | High | Low | High | Low | Low | **High** |
| Tug N 2020 | South-Africa | High | High | High | Low | Low | Low | Low | Low | Low | Low | **Moderate** |
| Vizheh M 2021 | Spain | High | High | High | Low | Low | Low | Low | Low | Low | Low | **Low** |
| Vouga  2021 | USA | High | High | High | Low | Low | Low | Low | Low | Low | Low | **Low** |
| Vousden N 2021 | Argentina | High | High | High | Low | Low | High | Low | Low | Low | Low | **Moderate** |
| WAPM 2020 | Turkey | Low | High | High | Low | Low | High | Low | Low | Low | Low | **Low** |
| Woodworth KR 2020 | Denmark | High | Low | High | Low | Low | High | Low | Low | Low | Low | **Low or Moderate** |
| Wu X 2020 | Iran | Low | High | High | Low | Low | High | Low | Low | Low | Low | **Low** |
| Yan J 2020 | Egypt | High | High | High | Low | Low | High | Low | Low | High | Low | **High** |
| Yassa M 2021 | France | Low | Low | High | Low | Low | High | Low | Low | Low | Low | **Low** |
| Yazihan N 2021 | Japan | Low | Low | High | High | High | High | Low | High | Low | Low | **Moderate** |
| Youssef AM 2023 | Iran | High | High | High | Low | Low | High | Low | Low | Low | Low | **Moderate** |
| Zelini P 2022 | India | High | High | High | Low | Low | High | Low | Low | High | Low | **High** |
| Ziert Y 2022 | Pakistan | High | High | High | Low | Low | High | Low | Low | High | Low | **High** |

**Supplementary Table S4. CHARACTERISTICS OF INCLUDED STUDIES**

| **Study** | **Country** | **Study type** | **All women**  **N** | **COVID-positiveN** | **1st trimester**  **N** | **2nd trimester N** | **1^st^ or 2^nd^ trimester**  **N** | **Age mean (SD/range)** | **BMI mean (SD/range)** | **Smoking**  **N** | **Previous PL**  **N** |
| --- | --- | --- | --- | --- | --- | --- | --- | --- | --- | --- | --- |
| **Aabakke AJM 2023** | Denmark | Cohort |  | 111185 |  |  | 853 | 29 (26-33) | 24 (21-27) | 89 |  |
| **Abedzadeh-Kalahroudi M 2021** | Iran | Prospective | 56 | 56 | 4 | 8 | 12 | 31.6 (6.1) |  |  |  |
| **Adhikari EH 2020** | USA | Retrospective | 3374 | 252 | 4 | 16 | 20 | 27 (6.6) | 30.5 (7.2) |  |  |
| **Ahmad SN 2022** | India | Retrospective | 6468 | 117 | 28 | 25 | 53 | (20-40) |  |  |  |
| **Ajith S 2021** | India | Retrospective | 350 | 350 |  |  |  |  |  |  |  |
| **Akram EG 2021** | Iraq | Retrospective | 100 | 100 | 100 |  | 100 |  |  |  |  |
| **Al-Hajjar S 2022** | Saudi-Arabia | Prospective | 81 | 81 | 17 | 30 | 37 | 31.8 (5.3) |  |  |  |
| **Anand P 2021** | India | Not reported | 69 | 69 |  |  |  | 26.7 (4.5) |  |  |  |
| **d'Antonio F 2021** | USA | Retrospective | 887 | 887 |  |  |  | 32.0 (5.8) |  | 32 |  |
| **Arinkan SA 2021** | Turkey | prospective | 116 | 46 | 5 | 13 | 18 | 28.9 (4.7) |  |  |  |
| **Arakaki T 2022** | Japan | Retrospective |  | 1043 | 202 | 380 | 461 | (30) | (26-34) |  |  |
| **Ayed A 2020** | Kuwait | Retrospective | 185 | 185 | 21 | 64 | 85 | 31 (27.5-34) |  |  |  |
| **Babic I 2022** | Saudi-Arabia | Retrospective |  | 209 | 3 | 15 | 18 | 31 (6.1) | 32.5 (6.6) |  |  |
| **Balachandren N 2022** | UK | Retrospective |  | 77 | 77 |  | 77 | 31 (27-39) | 24 (23.3) | 1 |  |
| **Barris M 2022** | Argentina | Not reported |  | 103 | 10 | 28 | 38 | 28 (15-42) |  |  |  |
| **Basu JK 2021** | South Africa | Retrospective | 103 | 103 | 25 | 63 | 88 | 30 (5.7) |  |  |  |
| **Bhoora S 2022** | South Africa | Retrospective | 204 | 204 | 11 | 31 | 42 | 31 (26-35) | 29.8 (26.7-36.1) |  |  |
| **Burwick RM 2021** | USA | Not reported | 67 | 67 |  |  |  | 33 (21-43) |  |  |  |
| **Çakirca TD 2021** | Turkey | Retrospective | 75 | 75 | 12 | 20 | 32 | 29 (18-45) |  |  |  |
| **Calderón JM 2020** | Mexico | Prospective | 20 | 20 |  |  |  | 25.5 (4.6) | 27.8 (5.1) |  |  |
| **Cambou M 2022** | USA | Prospective |  | 256 | 36 | 104 | 140 | 32 (25-35) |  |  |  |
| **Cardona-Pérez JA 2021** | Mexico | Retrospective | 70 | 70 |  |  |  | 26 (13-45) | 29 (19-57) | 1 |  |
| **Borges-Charepe N 2022** | Portugal | prospective | 630 | 630 | 35 | 73 | 108 | 30.0 (5.94) | 27 (5.9) |  |  |
| **Chaudhary S 2021** | Pakistan | Not reported |  | 41 | 2 | 8 | 10 | 27.9 (4.5) |  |  |  |
| **Chen L 2020** | China | Not reported | 118 | 118 | 22 | 21 | 43 | 31 (28-34) |  |  |  |
| **Chen S 2021** | USA | Not reported | 92 | 92 |  |  |  | 30 (19-42) | 32(21-56) |  |  |
| **Chung Y 2022** | Korea | Retrospective | 257 |  | 67 | 85 | 152 | 34 (31-37) | 23.6 (21-27) |  |  |
| **Crovetto F 2021** | Spain | Prospective | 2225 | 317 | 141 |  | 141 | 33.1 (29-37) |  | 18 |  |
| **Cosma S 2021** | Italy | Prospective | 147 | 17 | 17 | 0 | 17 | 32.1 (4) | 22.3 (3.2) | 1 | 3 |
| **Curi B 2020** | USA | Retrospective | 33 | 33 | 2 | 5 | 7 |  |  |  |  |
| **Daclin C 2022** | France | Retrospective | 172 | 86 |  |  |  | 31.9 (4.60) |  | 3 |  |
| **Delahoy M 2020** | USA | Not reported | 598 | 598 | 14 | 61 | 75 | 29 |  | 13 |  |
| **Deng Q 2021** | China | Retrospective | 39 | 39 | 4 | 8 | 12 | 30 (27-32) |  |  |  |
| **Devi KP 2021** | India | Retrospective | 129 | 129 |  |  |  | 29 (5.9) |  |  |  |
| **Di Mascio D 2021** | Worldwide | Retrospective | 388 | 388 | 31 | 86 | 117 | 32.2 (6.1) |  | 54 |  |
| **Diouf AA 2020** | Senegal | Not reported | 9 | 9 | 1 | 4 | 5 | 27.7 (3) |  |  |  |
| **Donati S 2021** | Italy | prospective | 786 | 198 |  |  | 198 |  |  |  |  |
| **Edlow AG 2020** | USA | Prospective | 127 | 64 | 9 |  | 9 | 31.6 (5.6) |  | 1 |  |
| **Emeruwa UN 2020** | USA | Retrospective | 100 | 100 |  |  |  | 28.5 (17-47) | 28 (16.5-45) |  |  |
| **Erol SA 2021** | Turkey | Prospective | 96 | 60 | 20 | 20 | 40 | 28.5 (6) | 26.5 (5.8) |  |  |
| **ESHRE working group 2021** | Multiple countries | retrospective | 105 | 80 | 24 | 16 | 40 | 33.7 (6.1) |  |  |  |
| **Fallach N 2022** | Israel | 2753 | 2753 | 478 | 943 | 1421 | 28 | (25-33) |  |  |  |
| **Fan C 2021** | China | Not reported | 12 | 12 | 2 | 3 | 5 | 29.6 (3.2) | 23.4  (4.6) |  |  |
| **Foo SS 2021** | USA/Brazil | Prospective | 93 | 93 | 18 | 31 | 49 | 33 (16-44) |  |  |  |
| **Fox NS 2020** | USA | Not reported | 92 | 92 |  |  |  | 31.4 (6.5) |  |  |  |
| **Gajbhiye R 2021b** | India | Prospective | 4203 | 4203 |  |  |  |  |  |  |  |
| **Göklü M 2023** | Turkey | Retrospective |  | 96 | 16 | 14 | 30 |  |  | 11 |  |
| **Grandone N 2022** | Italy | prospective | 90 | 90 |  |  |  | 32 (17-46) | 28.3 (4.7) |  |  |
| **Grechukhina O 2020** | USA | Retrospective | 141 | 141 | 11 | 37 | 48 | 30 (25-34) |  | 19 |  |
| **Guo Y 2021** | China | Retrospective | 20 | 20 | 3 | 2 | 5 | 30.15 (4.13) |  |  |  |
| **Hamadneh J 2022** | Jordan | Descriptive | 530 | 530 | 174 | 152 | 326 |  |  | 38 |  |
| **Haye MT 2021** | Chile | Prospective | 458 | 458 |  |  |  | 29.04 (6.38) |  |  |  |
| **Hazari KS 2021** | Dubai | retrospective | 79 | 79 | 17 | 15 | 32 | 32.7 (5.5) | 29.4 (5.5) |  |  |
| **Hcini N 2021** | French Guinea | Prospective | 507 | 137 |  |  |  | 25 (21-31) | 26.1 (22.3-30.8) |  |  |
| **Hernandez BO 2020** | Chile | Not reported | 661 | 661 | 52 | 119 | 171 | 29.9 | 30.4 (26.7-34.5) |  |  |
| **Hernandez PV 2023** | USA | Retrospective |  |  | 8 | 16 | 24 |  |  |  |  |
| **Hughes BL 2023** | USA | Retrospective |  | 2326 | 44 | 358 | 402 | 29 (5.9) | 29.3 (24.5-35.5) | 16 |  |
| **Jacoby VL 2021** | USA | Prospective | 94 | 94 |  |  |  | 31.2 (4.77) | 26.8 (6.5) | 1 |  |
| **Jang WK 2021** | Korea | retrospective | 7 | 7 | 4 | 1 | 5 | 34.57 (22-40) |  |  |  |
| **Kayem G 2020** | France | Not reported | 617 | 617 |  |  |  | * |  | 16 |  |
| **Khoiwal K 2021** | India | Prospective | 60 | 60 | 4 | 3 | 7 | 27.82 (5.10) |  |  |  |
| **Kiremitli S 2022** | Turkey | Retrospective | 52 | 52 | 52 |  | 52 | 29.4 (5.4) | 24.0 (3.8) |  |  |
| **Kumari A 2022** | India | Retrospective |  |  | 9 | 12 | 21 | 26 (5.2) |  |  |  |
| **Kuzan TY 2021** | Turkey | retrospective | 55 | 55 | 8 | 24 | 32 | 29.7 (6.4) |  |  |  |
| **Lei D 2020** | China | retrospective | 9 | 9 | 0 | 4 | 4 | 29 (24-35) |  |  |  |
| **Lokken EM 2021** | USA | retrospective | 240 | 240 | 38 | 67 | 105 | 28 (24-34) |  |  |  |
| **London V 2020** | USA | Retrospective | 55 | 55 | 0 | 1 | 1 | 31 (23.5-34.8) | 31 (36.3-34.5) |  |  |
| **Mahajan NN 2021** | India | Retrospective | 879 | 879 |  |  |  | 27 (24-34) |  |  |  |
| **Manasova G 2021** | Ukraine | retrospective | 218 | 218 | 15 | 56 | 81 | 29.1 (6.5) | 39 obese |  |  |
| **Martinez-Baladejo MT 2023** | China | Retrospective | 47 | 47 | 1 | 19 | 20 | 30.2 (5.3) | 64%>30 | 4 |  |
| **Martínez Varea A 2022** | Spain | Retrospective | 487 | 487 | 59 | 149 | 208 | 32.0 (5.5) |  | 32 |  |
| **Mattar CN 2020** | Singapore | Prospective | 16 | 16 | 6 | 1 | 7 | 23 (25-36) |  |  |  |
| **McCreary EK 2022** | USA | Retrospective | 1140 | 944 | 143 | 371 | 514 | 30 (26-33) |  |  |  |
| **Metkari AM 2020** | India | Retrospective |  | 107 | 2 | 4 | 6 |  |  |  |  |
| **Metz TD 2021** | USA | prospective | 14104 | 2352 | 54 | 414 | 468 | 28 (6.2) | 30 (7.8) | 4.3% |  |
| **Mullins E 2021** | PAN-COVID | Registry | 3999 | 3999 |  |  |  | (28.6-32) |  |  |  |
| **Nambiar SS 2020** | India | Retrospective | 158 | 158 |  | 42 | 42 | * |  |  |  |
| **Neelam V 2023** | USA | Retrospective | 35200 |  | 6458 |  | 6458 |  |  |  |  |
| **Omrani AS 2020** | Qatar | Retrospective | 26 | 26 | 3 | 10 | 13 | 29 (25-33) |  |  |  |
| **Overtoom EM 2021** | The Netherlands | Prospective | 376 | 376 | 49 | 101 | 150 |  |  | 16 |  |
| **Péju E 2022** | European | Retrospective |  | 2480 | 14 | 71 | 85 | 33 (6) | 29.1 (26.4-33.5) |  |  |
| **Pirjani R 2020** | Iran | Prospective | 199 | 66 |  | 17 | 17 | 31 (6.4) | 29.5 |  |  |
| **Poisson M 2023** | France | Retrospective |  | 501 | 22 | 120 |  |  |  | 35 |  |
| **Poon LC 2021** | Hong Kong | Prospective | 23 | 23 |  |  |  | 35.5 (31-38.8) |  |  |  |
| **Priyadharshini C 2021** | India | Not reported | 381 | 381 | 7 | 18 | 25 | 25.98 (4.35) |  |  |  |
| **Qiancheng X 2020** | China | Retrospective | 28 | 28 | 3 | 1 | 4 | 30 (27-32) |  |  |  |
| **Qudsieh S 2022** | Jordan | Retrospective | 112 | 112 | 6 | 18 | 24 | 30 (5.4) |  |  |  |
| **Regan A 2022** | USA | retrospective | 7803 | 2515 |  |  |  |  |  |  |  |
| **Rozo N 2021** | Colombia | Retrospective | 5614 | 5614 |  | 456 | 456 | (15-44) |  |  |  |
| **Sahin D 2021** | Turkey | Prospective | 1416 | 1416 | 311 | 433 | 744 | 28.47 (5.63) | 26.8 (5.3) |  | 0 |
| **Sahin D 2022** | Turkey | prospective | 1935 | 519 |  |  |  | 29.4 (5.55) |  |  |  |
| **Saimin J 2021** | Indonesia | Retrospective | 41 | 41 | 5 |  |  | 30 (19-39) |  |  |  |
| **Santhosh J 2020** | Oman | Retrospective | 60 | 60 | 6 | 6 | 12 | 32 (6) | 31 (6) |  |  |
| **Santos CAD 2022** | Brasil | Prospective | 88 | 84 | 20 | 43 | 63 | 29.9 (10) |  |  |  |
| **Schell RC 2022** | USA | Prospective | 1326 |  | 103 | 355 | 458 | 28.5 (6.6) |  |  |  |
| **Sekkarie A 2022** | USA | Retrospective |  | 336 | 30 | 65 | 95 | (15-49) |  |  |  |
| **Sentilhes L 2020** | France | Retrospective | 54 | 54 | 1 |  | 1 | 30.6 (6.2) | 25.3 (6.7) |  |  |
| **Sertel E 2023** | Turkey | Retrospective | 208 | 208 | 30 | 48 | 78 | 28 (25-33) |  |  |  |
| **Shah PT 2020** | India | Prospective | 125 | 125 | 10 | 10 | 20 | 25.2 (18-40) |  |  |  |
| **Shmakov RG 2020** | Russia | Prospective | 66 | 66 | 5 | 7 | 12 | 30.3 (6.3) | 27.1 (4.6) |  |  |
| **Singh V 2021** | India | Retrospective | 132 | 132 | 5 | 2 | 7 | 27.5 (4.6) |  |  |  |
| **Souza RT 2022** | Brasil | Prospective | 729 | 289 | 36 | 73 | 109 | Majority 20-35 |  | 2 |  |
| **Sunder A 2022** | Bahrein | retrospective | 74 | 74 | 16 | 10 | 26 | 29.6 (5.9) | 32 (6) |  |  |
| **Taghavi SA 2021** | Iran | Retrospective |  | 55 | 10 | 7 | 17 | 30.5 (4.2) | 26.1 (3.4) |  |  |
| **Takemoto ML 2020** | Brazil | retrospective | 20 | 20 | 2 | 1 | 3 | 31.5 (20-43) |  |  |  |
| **Tanacan A 2021** | Turkey | Prospective | 180 | 90 | 30 | 30 | 60 | 28 (6) | 25.6 (4.5) |  |  |
| **Tavakoli N 2023** | Iran | Retrospective |  | 189 | 7 | 20 | 27 |  |  |  |  |
| **Tug N 2020** | Turkey | Retrospective | 188 | 188 | 37 | 68 | 105 | 31 (12) |  |  |  |
| **Vizheh M 2021** | Iran | Retrospective | 110 | 110 | 6 | 32 | 38 | 32.02 (6.1) |  |  |  |
| **Vouga M 2021** | Switzerland | Prospective | 926 | 926 |  |  |  | 32 (28-36) | 26 (23-30) | 61 |  |
| **Vousden N 2021** | UK | prospective | 4436 | 4436 |  |  | 455 |  |  | 99 |  |
| **WAPM 2021** | World | Retrospective | 388 | 388 | 31 | 86 | 117 | 30.6 (9.5) | 7.2% obese | 13.9% smoker |  |
| **Woodworth KR 2020** | USA | Retrospective | 4442 | 4442 | 13 | 502 | 515 | 28.9 (22.4-34.0) |  |  |  |
| **Wu X 2020** | China | Retrospective | 23 | 23 | 3 | 0 | 3 | (21-36) |  |  |  |
| **Yan J 2020** | China | Retrospective | 116 | 116 | 4 | 6 | 10 | 30.8 (24-41) |  |  |  |
| **Yassa M 2021** | Turkey | Retrospective | 8 | 8 | 3 | 3 | 6 | 32.5 (19-41) |  |  |  |
| **Yazihan N 2021** | Turkey | Prospective | 187 | 95 | 32 | 32 | 64 | 29 (7) | 26.6 (5.5) |  |  |
| **Youssef AM 2023** | Egypt | Retrospective |  | 46 | 3 | 3 | 6 | 30.4 (6.1) | 23.0 (2.2) |  |  |
| **Zelini P 2022** | Italy | Prospective |  | 48 |  |  | 12 | 28 (18-45) |  |  |  |
| **Ziert Y 2022** | Germany | Prospective | 1485 | 1485 | 196 | 481 | 677 | 31 (5.1) |  |  |  |

* Majority below 35
Nr = Number; Nr total = Number total; Nr COVID pos = number of confirmed COVID cases; SD = standard deviation; ntot = total number first and second trimester pregnancies; BMI = Body mass index; PL = pregnancy loss; wk = week; wks; = weeks; EP = Ectopic pregnancy

**Supplemetary Table S5. Definitions of pregnancy loss**

| S**tudy** | Definition |
| --- | --- |
| **Aabakke A 2023** | Abortion; 1 inevitable abortion and incomplete abortion, 2 missed abortion); Ectopic pregnancy |
| **Aabakke AJM 2021** | No definition |
| **Abedzadeh-Kalahroudi M 2021** | Induced abortion in 1st trimester and 2nd trimester |
| **Adhikari E H 2020** | No definition |
| **Ahmad SN 2022** | Miscarriage; not defined; GA between 8-19 weeks |
| **Ajith S 2021** | Pregnancy loss defined < 20 weeks |
| **Akram E G 2020** | Pregnancy loss defined <24 weeks |
| **Al-Hajjar S 2022** | Miscarriage defined at < 20 weeks gestation |
| **Anand P 2021** | Pregnancy loss; 7 in first trimester (mean gestational age 7 weeks); 7 in second trimester (mean gestational age 17 weeks) |
| **Arakaki T 2022** | Pregnancy loss defined as <20 weeks |
| **Arinkan S A 2021** | No definition |
| **Ayed A 2020** | Miscarriage defined <20 weeks of GA |
| **Babic I 2022** | No definition |
| **Balachandren N 2022** | No definition |
| **Barris M 2022** | No definition |
| **Basu JK 2021** | No definition |
| **Bhoora S 2022** | Miscarriage defined <14 weeks of GA |
| **Borges-Charepe N 2022** | Miscarriage defined <20 weeks of GA |
| **Burwick R M 2020** | No definition |
| **Çakirca TD 2021** | Pregnancy loss < 20 weeks of GA |
| **Calderón J M 2020** | Abortion <24 weeks of GA |
| **Cambou M 2022** | No definition |
| **Cardona-Pérez J A 2021** | Miscarriages; not defined; GA 5 <14 weeks; 1 14-22; 1 molar (< 12wk) |
| **Chaudhary S 2021** | Pregnancy loss defined < 22 weeks of GA |
| **Chen L 2020** | Pregnancy loss defined < 20 weeks of GA |
| **Chen S 2021** | Abortion; GA at 7 weeks |
| **Chung Y 2022** | Spontaneous abortion; not defined |
| **Cosma S 2021** | Pregnancy loss defined <22 weeks of GA |
| **Crovetto F 2021** | Abortion; 1st and 2nd trimester |
| **Curi B 2020** | Missed abortion; no definition |
| **Daclin C, 2022** | Late miscarriage- pregnancy loss at <24 WG |
| **d'Antonio F 2021** | In first of second - spontaneous abortion and induced abortion not defined |
| **Delahoy M J 2020** | abortion (Pregnancy loss) <21 weeks |
| **Deng Q 2021** | spontaneous abortion; no definition |
| **Devi KP 2021** | miscarriage; no definition |
| **Di Mascio D 2021** | Pregnancy loss defined as <22 weeks of GA(39 1 st+9 2nd), termination of pregnancy |
| **Diouf AA 2020** | spontaneous abortion; no definition |
| **Donati S, 2021** | Abortus per trimester; no definition |
| **Edlow A G 2020** | Miscarriage; no definition |
| **Emeruwa UN 2020** | Miscarriage <24 weeks |
| **Erol S A 2021** | Abortion; no definition |
| **ESHRE working group 2021** | Abortion; no definition, but GA 7 and 18 weeks |
| **Fallach N 2022** | Miscarriage was defined as pregnancy loss before 22 weeks |
| **Fan C 2021** | Miscarriage; no definition, however GA: 14, 13, and 14 weeks |
| **Foo SS 2021** | Early miscarriage, defined as PL <13 weeks |
| **Fox N 2020** | Miscarriage; not defined |
| **Gajbhiye RK 2021** | First trimester loss, <14 weeks. 14 in total 1 excluded because it was a molar pregnancy --> 13 in analysis |
| **Göklü M 2022** | Spontaneous miscarriage; not defined but GA 17 weeks |
| **Grandone N 2022** | Spontaneous abortion between 8 and 14 weeks |
| **Grechukhina O 2020** | Fetal loss: no definition, but 1x GA 16 weeks and 1x ectopic pregnancy |
| **Guo Y 2021** | Miscarriage; not defined |
| **Hamadneh J 2022** | spontaneous abortion; no definition |
| **Haye M T 201** | Miscarriage; not defined but GA 19 weeks |
| **Hazari K S 2021** | Miscarriage; not defined |
| **Hcini N 2020** | Abortion; no definition |
| **Hernandez B O 2020** | Miscarriage; no definition |
| **Hernandez P V 2023** | Miscarriage; no definition |
| **Hughes BL 2022** | Pregnancy loss < 20 wks of gestation. Might include therapeutic abortion. |
| **Jacoby 2021** | Spontaneous abortion; no definition, but GA 6 weeks |
| **Jang WK 2021** | Spontaneous abortion, no definition, but GA 7, 7 and 18 weeks |
| **Kayem G 2020** | Miscarriage defined <22 weeks |
| **Khoiwal K 2021** | Miscarriage, no definition, but GA 7 weeks |
| **Kiremitli S 2022** | Miscarriage, no definition |
| **Kumari A 2022** | No definition |
| **Kuzan T Y 2021** | Spontaneous abortion; no definition |
| **Lei D 2020** | Miscarriage; no definition |
| **Lokken E M 2021** | Miscarriage; no definition |
| **London V 2020** | No definition, 2 induced abortion GA 21 and 17 weeks |
| **Mahajan N N 2021** | Miscarriage defined PL < 20 weeks |
| **Manasova G 2021** | Fetal demise; no definition but GA at 14 weeks |
| **Martínez Varea A 2022** | Spontaneous abortion < 20 weeks |
| **Martinez-Baladejo MT 2023** | Miscarriage defined <20 weeks |
| **Mattar C N 2020** | Spontaneous abortion; not defined but in 1st trimester |
| **McCreary EK 2022** | spontaneous abortion,, no definition |
| **Metkari AM 2020** | Miscarriage; no definition, GA ranging 7 to 15 |
| **Metz T D 2022** | Spontaneous miscarriage; not defined but first trimester miscarriage |
| **Mullins E 2021** | Termination of pregnancy |
| **Nambiar S S 2020** | Abortion |
| **Neelam V 2022** | Early pregnancy loss defined <20 weeks of GA |
| **Omrani AS 2020** | Miscarriage; no definition |
| **Overtoom E M 2021** | Early pregnancy loss defined as aboriton <20 weeks POG |
| **Péju E 2022** | Fetal loss 14-21 weeks |
| **Pirjani R 2020** | Missed abortion < 7 weeks (1 case) |
| **Poisson M 2023** | Pregnancy loss <24 weeks of GA |
| **Poon L C 2021** | Spontaneous abortion in 1st trimester pregnancy |
| **Priyadharshini C 2021** | No definition; 1 fetal demise at GA 17 weeks |
| **Qiancheng X 2020** | Spontaneous abortion; no definition |
| **Qudsieh S 2022** | Spontaneous abortion; GA 13-17 weeks |
| **Regan A, 2022** | Miscarriage defined as GA <24 weeks |
| **Rozo N 2021** | Miscarriage defined as GA <20 weeks |
| **Sahin D 2021** | Miscarriage; GA not defined |
| **Sahin D 2022** | Miscarriage; all reported <20 weeks |
| **Saimin J 2021** | Miscarriage; not defined; |
| **Santhosh J 2020** | Miscarriage; not defined |
| **Santos CAD 2022** | Spontaneous miscarriage; not defined |
| **Schell RC 2022** | Abortion; not defined |
| **Sekkarie A 2022** | Abortion is defined as spontaneous or induced termination of pregnancy before fetal viability |
| **Sentilhes L 2020** | Medical abortion |
| **Sertel E 2023** | Abortion <24 weeks |
| **Shah PT 2020** | Pregnancy loss; not defined |
| **Shmakov R G 2020** | No defintion |
| **Singh V 2021** | No definition |
| **Souza RT 2022** | Miscarriage |
| **Sunder A 2022** | Spontaneous miscarriage; not defined |
| **Taghavi S 2021** | Miscarriage; not defined |
| **Takemoto MLS 2020** | Missed abortion; not defined GA 10, 14, 18 weeks |
| **Tanacan A 2021** | Spontaneous abortion; GA 12, 18 19 weeks + 1 medical abortion. |
| **Tavakoli N 2022** | Spontaneous miscarriage; not defined but in 1st trimester |
| **Tug N 2020** | Miscarriage not defined |
| **Vizheh M 2021** | Spontaneous miscarriage; not defined; GA not reported, 13 and 22 weeks |
| **Vouga M 2021** | Miscarriage; not defined |
| **Vousden N 2021** | Abortion; GA 7 weeks |
| **Woodworth KR 2020** | Spontaneous abortion; no definition |
| **Wu X 2020** | Late miscarriage defined as GA 14-24 weeks |
| **Yan J 2020** | Pregnancy loss; not defined |
| **Yassa M 2021** | Miscarriage is defined as <22 weeks of GA |
| **Yazihan N 2021** | Abortion |
| **Youssef A M 2023** | Spontaneous abortion in early 2nd trimester |
| **Zelini P 2022** | Missed abortion |
| **Ziert Y 2022** | Miscarriage; not defined |

Supplementary Figure S1. Proportion of miscarriage in pregnant women with SARS-CoV-2 infection in all studies with data on miscarriage during the first and/or second trimester


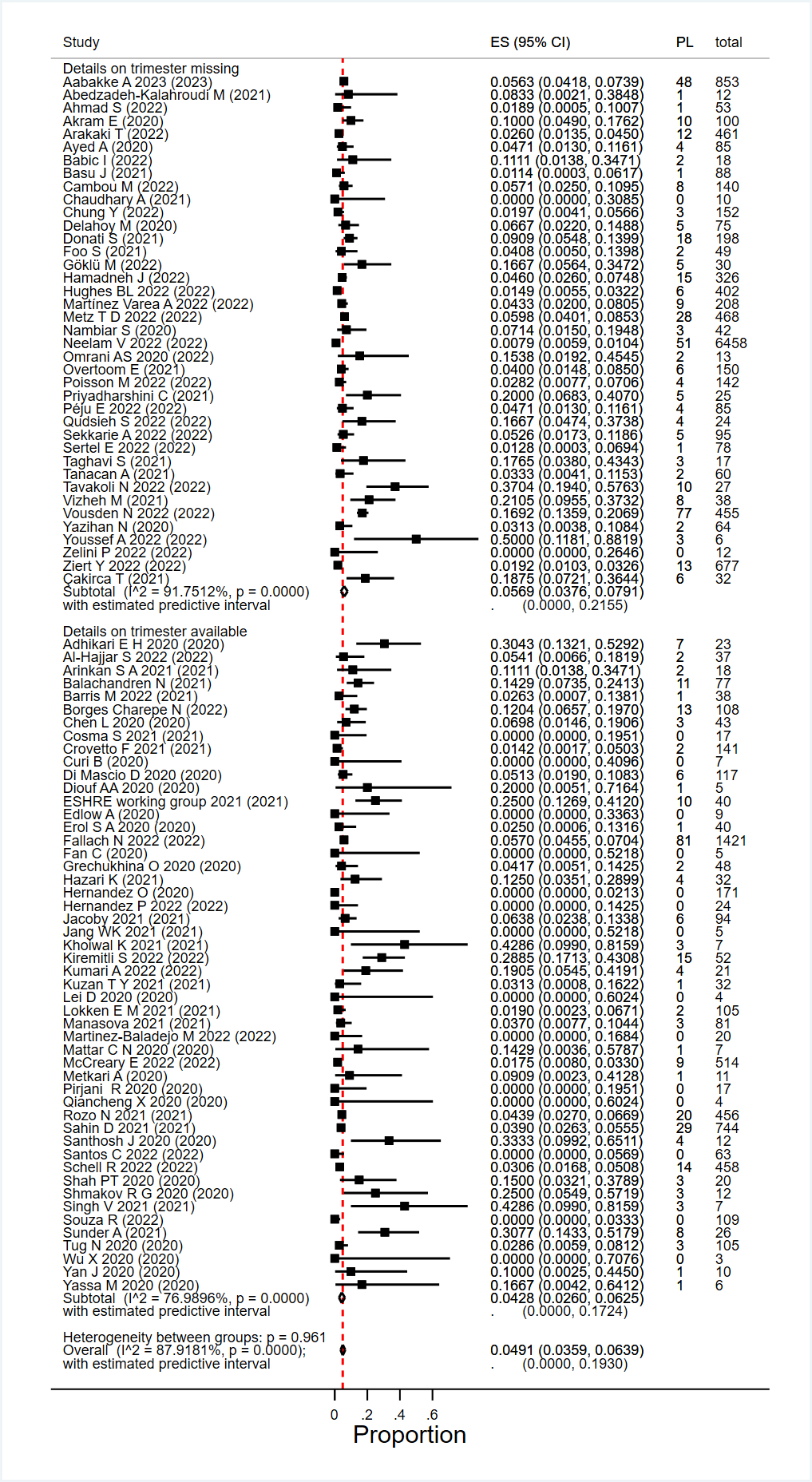

Supplement: dmad030_Supplementary_Data [file dmad030_supplementary_data.docx]
